# Is the apparently protective effect of maternal nicotine replacement therapy (NRT) used in pregnancy on infant development explained by smoking cessation?: secondary analyses of a randomised controlled trial

**DOI:** 10.1136/bmjopen-2018-024923

**Published:** 2019-07-11

**Authors:** Barbara Iyen, Luis R Vaz, Jaspal Taggar, Sue Cooper, Sarah Lewis, Tim Coleman

**Affiliations:** 1 Division of Primary Care, University of Nottingham, Nottingham, UK; 2 Division of Epidemiology and Public Health, University of Nottingham, Nottingham, UK

**Keywords:** smoking in pregnancy, maternal smoking, child development, developmental impairment

## Abstract

**Objective:**

To investigate relationships between maternal smoking status in pregnancy and infant development. The largest randomised controlled trial of nicotine replacement therapy (NRT) for smoking cessation in pregnancy, the smoking, nicotine and pregnancy (SNAP) trial, found that at 1 month after randomisation, smoking cessation rates were doubled in the NRT group compared with the placebo group. At delivery, there was no significant difference in cessation rates between groups. Surprisingly, infants born to women randomised to NRT were more likely to have unimpaired development at 2 years. We hypothesised that this apparently protective effect was due to smoking cessation caused by NRT and so, investigate this relationship using the same cohort.

**Design:**

Secondary analysis of a randomised controlled trial.

**Setting:**

Seven antenatal hospitals in the Midlands and North-West England.

**Participants:**

Eight hundred and eighty-four pregnant smokers randomised to receive either NRT patches or visually-identical placebo in the SNAP trial. Participants’ smoking behaviour were recorded at randomisation, 1 month after their target quit date and at delivery.

**Methods:**

Using logistic regression models, we investigated associations between participants’ smoking measures and infant development (assessed using the Ages and Stages questionnaire) at 2 years.

**Main outcome measures:**

2 year infant development.

**Results:**

Developmental impairment was reported for 12.7% of study 2 year olds. Maternal heaviness of smoking at randomisation (OR: 1.26, 95% CI: 0.82 to 1.96, p=0.091), validated smoking abstinence recorded at 1 month after a quit date (OR: 1.02, 95% CI: 0.60 to 1.74, p=0.914) and validated smoking abstinence recorded at both 1 month after a quit date and at the end of pregnancy (OR: 1.52, 95% CI: 0.81 to 2.85, p=0.795) were not independently associated with infant developmental impairment at 2 years.

**Conclusion:**

We found no evidence that NRT treatment improved infants' developmental outcomes through smoking cessation.

**Trial registration number:**

CTA03057/0002/001-0001; Post-results

**Strengths and limitations of this study**

- This study uses data from the smoking, nicotine and pregnancy (SNAP) trial, the largest randomised controlled trial of nicotine replacement therapy for smoking cessation in pregnancy.
- The SNAP trial remains the only maternal smoking cessation trial to investigate infant outcomes beyond delivery.
- Baseline characteristics and potential confounders were included in multivariable analyses to determine independent associations between maternal smoking and 2 year developmental outcomes.
- Maternal factors which were not measured in the SNAP trial could not be adjusted for in multivariable analyses.

## Introduction

Smoking in pregnancy is associated with increased risks of many adverse outcomes including miscarriage, stillbirth, prematurity, low birth weight, perinatal morbidity and mortality^1^ and is a substantial international public health problem; in high income countries 13% to 25% of pregnant women smoke^2–5^ and in developing countries,^6 7^ the WHO predicts a future epidemic. Fortunately, impacts on the foetus are avoidable and cessation in pregnancy improves infants’ birth weights and reduces the risk of premature birth.^8^ Additionally, pregnancy is the life event which seems to most motivate smokers’ cessation attempts; over 50% of UK pregnant smokers try stopping.^5^ Women are, therefore, likely to be receptive to support with stopping and, as it is effective for non-pregnant smokers^9^ and possibly also for pregnant smokers too, nicotine replacement therapy (NRT) is frequently offered to pregnant women who smoke.^10^ Although NRT is not thought to be completely risk-free because it exposes users to nicotine, this is considered safer than smoking because smokers are already nicotine-exposed and NRT users are not exposed to the numerous harmful chemicals and tobacco smoke carcinogens such as carbon monoxide, tar and lead.

Animal experiments and human-subject laboratory investigations suggest that nicotine may adversely affect the foetal cardiovascular system and developing nerve tissue,^11 12^ however trials of NRT for smoking cessation in pregnancy have been permitted because any foetal harm from the nicotine would likely be compensated for by the benefits of mothers stopping smoking.^13^ In the largest of these trials, compliance rates in both the NRT and the placebo arms were low, with only 7.2% of women assigned to receive NRT and 2.8% of women assigned to receive placebo, using patches for more than 1 month. Nicotine patches doubled cessation rates at 4 weeks but by delivery there was no significant difference in cessation rates between the NRT and placebo groups.^14^ Unexpectedly, infants born to NRT group women were 40% more likely to have unimpaired development at 2 years of age than infants born to women in the placebo groups^15^ (OR: 1.40, 95% CI: 1.05 to 1.86, p=0.023) and there was a dose-response relationship between adherence to NRT and impairment-free infant development.^15^ It seems implausible that this effect would be due to nicotine having a direct beneficial effect on the foetus, and there is a possibility that the observed effect might be due to differences in unmeasured characteristics between the participants in the NRT group and those in the placebo group. However, as NRT had a substantial impact on cessation in early pregnancy, we hypothesise that the better developmental outcomes in NRT group infants and the dose-response relationship between increasing NRT use and these might be explained by smoking cessation during pregnancy caused by NRT use. Consequently, we present secondary analyses of data from the same trial to investigate whether the absence of infants’ developmental impairments at 2 years was associated with maternal smoking status measured at different points in the trial.

## Methods

### Study participants

We used data from the smoking, nicotine and pregnancy (SNAP) trial, a placebo randomised-controlled trial of NRT for smoking cessation in pregnant women.^14^ The trial recruited 1050 participants aged 16 to 45 years between 12 and 24 weeks gestation who smoked at least 10 cigarettes a day before pregnancy and at least five cigarettes a day during pregnancy, with exhaled carbon monoxide (CO) readings of ≥8 ppm. Participants were randomised to receive up to an 8 week course of either 15 mg per 16 hours of NRT patches or visually-identical placebo. For safety reasons, women were instructed that if they smoked at all while using patches, the NRT should be stopped. Participants set a target quit date and were followed up 1 month after this, during hospital admission for delivery or as soon as possible afterwards and at 24 months after delivery.

### Maternal baseline data and smoking behaviour measures

Baseline data included maternal date of birth, age on leaving full-time education, number of cigarettes smoked per day prior to and during pregnancy, Body Mass Index (BMI), the Heaviness of Smoking Index (HSI) - a measure of nicotine dependence which is a six-point scale derived from the time to first cigarette after waking and the number of cigarettes smoked daily.^16^ Other data included were previous use of NRT in the current pregnancy and partners’ smoking status.

Self-reported smoking data were obtained 1 month after the target quit date and at delivery. Self-reported smoking abstinence at 1 month after the quit date was validated by exhaled CO measurements below 8 ppm while abstinence at delivery was validated by CO measurements and saliva cotinine concentrations below 10 ng/mL. Carbon monoxide is exhaled from the breath after smoking cigarettes and was used to confirm abstinence within the preceding 24 hours. Salivary cotinine on the other hand was used to determine smoking exposure within the last 7 days.^17^ No data on tobacco smoke exposure were collected between the 1 month follow-up date and delivery. Self-reported data on the use of nicotine patches were collected 1 month after the target quit date and at delivery; adherence to NRT have been reported elsewhere.^14^

### Infant outcome measures

Birth status (live or stillbirth), gestational age at birth, birth weight, singleton or multiple birth, sex and gestational age at birth were recorded at delivery and have been reported elsewhere.^14^ The presence of infant developmental impairment at 24 months was determined using questionnaire responses from participants or from healthcare professionals when there was no participant response; full details have been described previously.^15^ Participants reported on their infants’ development as assessed within the five domains of the Ages and Stages Questionnaire, Third Edition (ASQ-3): communication, gross motor, fine motor, problem solving and personal-social skills.^15^ Participant questionnaires (PQ2) were posted out at 24 months and if no response was received, health professional ones were dispatched to non-respondents’ family physicians. Health professional questionnaire (HPQ) items were consistent with domains in participant questionnaires and were designed to be completed with reference to medical or health visitor records. Health professionals completing these questionnaires required relatively little knowledge of the patient. However if they were unable to complete the questionnaire, they were asked to forward these to children’s’ health visitors.^18^ Scores from the five ASQ-3 domains and responses to ‘non-domain’ ASQ-3 items in the participant questionnaires were used with established norms to categorise infants as having ‘developmental impairment’ or ‘no developmental impairment’. Infants with only HPQ responses were considered to have no developmental impairment when the responses from all questions indicated no potential developmental issues.^15^ As shown in the main study, 88% of study participants returned a PQ2 or HPQ. The proportion of participants assessed using the PQ2 or HPQ were similar in the treatment arm and placebo arm. Approximately two-thirds of participants completed and returned the PQ2, while the HPQ was the source of data for children of the remaining participants.

### Measures of tobacco smoke exposure

As outlined above, smoking behaviour measures were collected at randomisation, 1 month after a target quit date and at the end of pregnancy. From these data, we derived the following categories to represent different levels of tobacco smoke exposure during pregnancy: (i) Number of cigarettes smoked daily during pregnancy, at randomisation, (ii) HSI at randomisation, categorised as high (4 to 6) or low (0 to 3), (iii) Women who reported not smoking between their target quit date and 1 month, validated using exhaled CO and (iv) Women who reported not smoking between their target quit date and the end of pregnancy or shortly afterwards, validated by either or both of exhaled CO and saliva cotinine at the end of pregnancy and also by exhaled CO at 1 month after their quit date.

As all trial participants smoked at the time of randomisation, exposure category (ii) was intended to dichotomise those who smoked more heavily and less heavily in the first weeks of pregnancy prior to study randomisation. For categories (iii) and (iv), we hypothesised that those demonstrating validated abstinence at both 1 month and the end of pregnancy (category iv) would have a lower overall tobacco smoke exposure than those for whom abstinence was only validated at 1 month after their quit date. However, it should be noted that no biochemical test can validate smoking cessation for a prolonged period and some women in either group may have smoked for a time between baseline/study enrolment and 1 month or the end of pregnancy.

### Analyses

Maternal and infant characteristics were analysed by study arm, for participants who had singleton live births and 2 year data on infant developmental impairment. Summary statistics were represented as number (%), mean (SD) and median (IQR) for categorical, normal continuous and non-normal continuous variables, respectively. Univariable logistic regression investigated the association between 2 year developmental outcomes and different measures of maternal tobacco smoke exposure such as baseline number of cigarettes smoked daily in pregnancy, the heaviness of smoking index during pregnancy, maternal smoking abstinence at some point during pregnancy (measured 1 month after the participants’ set quit date) and maternal smoking abstinence during the latter stages of pregnancy (measured at delivery). In multivariable analyses, we adjusted the univariate models for baseline characteristic variables and also controlled for variables that could confound the association between maternal smoking and infant developmental impairment. Potential confounders included in the multivariable analyses were: maternal age, age at which full-time education was completed, maternal BMI, partner’s smoking status, infant birth weight, gestational age at birth and study arm (NRT or placebo). Confounder selection was done using the change-in-estimate criteria,^19^ and any covariate which changed the effect size of the univariate exposure-outcome model by 10% was considered an important confounder and included in the fully-adjusted model. All analyses were performed using Stata V.14. Significant associations were defined at the p≤0.05 significance level.

### Patient and public involvement

The original SNAP study had a patient and public involvement (PPI) representative who provided PPI insights for the Trial Steering Committee.

## Results

**Figure 1:**
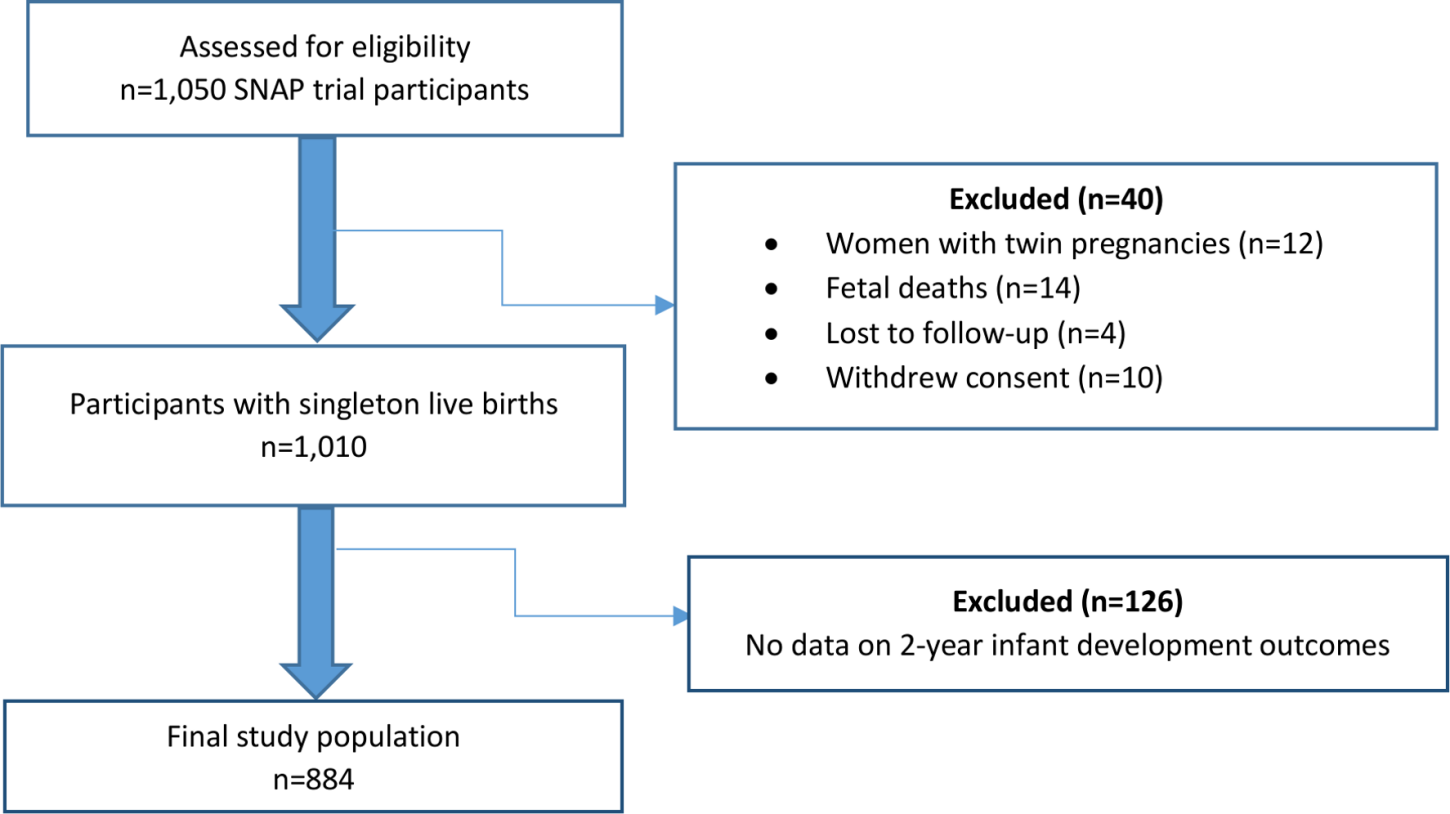
Flow diagram of study participants. Participants with twin pregnancies (n=12), foetal deaths (n=14), loss to follow-up (n=4) and those who withdrew consent (n=10) were excluded from the study analyses. Among participants with singleton live births, there were no data on 2 year infant outcomes for 126 participants, so these participants were also excluded from the analyses. SNAP, smoking, nicotine and pregnancy.

From 1050 pregnant smokers enrolled in the SNAP trial, there were 12 women with twin pregnancies, 14 participants who had foetal deaths, four lost to follow-up and 10 who withdrew consent. Of 1010 women with singleton live births, data on developmental impairment was available for 884 infants. Analyses therefore included maternal and 2 year records of these 884 infants (figure 1). The self-reported median (IQR) number of daily cigarettes smoked by women prior to pregnancy was 19 (10 to 60). At the time of recruitment into the study, the reported median (IQR) number of cigarettes smoked daily in pregnancy was 15 (10 to 20). Of the infants born to participants in the study, 52% were males and less than 10% had low birth weight. Developmental impairment was present in 12.7% of these children (n=112).

Study participants and singleton infants with 2 year outcome data had similar baseline characteristics to participants reported in the full trial cohort.^15^ Aside from the fact that participants who were lost to follow-up had higher mean index of multiple deprivation scores implying that they lived in more deprived areas than participants who had data up to 2 years, there were only minor differences in the baseline characteristics between the participants with 2 year data and those who were lost to follow-up.^15^

### Maternal and infant characteristics

The baseline characteristics of the women and children included in the analyses were found to be similar in participants randomised to the NRT or placebo study arm (presented in table 1). The majority of participants included in the study (72.4%) were less than 30 years old; the median BMI (IQR) at randomisation into the study was 25.9 (22.2 to 30.7). The average age of leaving full-time education was 16.3 years. We used the ‘Heaviness of Smoking Index’ as a measure of the level of maternal nicotine dependence and 65% of women in the study had low levels of nicotine dependence prior to being recruited in the study. More than two-thirds of the study participants had partners who smoked (67.9%). Overall, 18% of women were validated as abstinent from smoking at some point during pregnancy (measured 1 month after their target quit date) and 8.9% remained abstinent at the latter stages of pregnancy until delivery. There was a statistically significant difference in smoking cessation outcomes between participants in the NRT and placebo arms at the 1 month follow-up but not at delivery.

**Table 1:** Maternal and infant characteristics, by study arm (n=884)

	Unit	NRT arm (n=443)	Placebo arm (n=441)	P value
Maternal characteristics				
Maternal age (years) at pregnancy				
<20		72 (16.25)	64 (14.51)	
20–24		142 (32.05)	150 (34.01)	
25–29	n (%)	110 (24.83)	102 (23.13)	0.275
30–34		64 (14.45)	84 (19.05)	
35–39		44 (9.93)	30 (6.80)	
>40		11 (2.48)	11 (2.49)	
Maternal BMI (kg/m^2^) (n=847)	Median (IQR)	25.71 (22.2–30.7)	26.32 (22.5–30.8)	0.042
Index of multiple deprivation score	Mean (SD)	32.11 (16.83)	32.29 (16.84)	0.754
Maternal age (years) of leaving full-time education (n=872)	Mean (SD)	16.17 (1.36)	16.32 (1.70)	0.95
Partner’s smoking status				
Non-smoker		108 (24.38)	106 (24.04)	0.962
Smoker	n (%)	301 (67.95)	299 (67.80)	
No data on partner’s smoking status		34 (7.67)	36 (8.16)	
Heaviness of smoking index at study randomisation	n (%)			
Low index (0–3)		284 (64.11)	292 (66.21)	0.511
High index (4–6)		159 (35.89)	149 (33.79)	
Daily number of cigarettes smoked at study randomisation	Median (IQR)	13 (10–20)	15 (10-20)	0.799
Smoking cessation measured during pregnancy (at 1 month follow-up)	n (%)			
Did not cease smoking		341 (76.98)	384 (87.07)	<0.0001
Ceased smoking		102 (23.02)	57 (12.93)	
Maternal smoking cessation measured at time of delivery	n (%)			
Did not cease smoking		397 (89.62)	408 (92.52)	0.131
Ceased smoking		46 (10.38)	33 (7.48)	
Infant characteristics				
Infant sex				
Female	n (%)	217 (48.98)	205 (46.49)	0.457
Male		226 (51.02)	236 (53.51)	
Birth weight				
<2.5kg		48 (10.84)	37 (8.39)	
2.5–3.0kg	n (%)	111 (25.06)	104 (23.58)	0.534
3.0–3.5kg		161 (36.34)	174 (39.46)	
>3.5kg		123 (27.77)	126 (28.57)	
Gestational age at birth (weeks)	Median (IQR)	39.5 (2.1)	39.5 (2.2)	

BMI, Body Mass Index; NRT, nicotine replacement therapy.

Analyses of the associations between different measures of maternal tobacco smoke exposure and infant developmental impairment found no statistically significant relationship between maternal smoking exposure and infant developmental impairment at age 2 (table 2). The ORs for developmental impairment in infants of mothers with validated smoking cessation at some point during pregnancy (measured at the 1 month follow-up) and in infants of mothers with validation cessation at delivery, were 0.99 (95% CI 0.59 to 1.66) and 1.55 (95% CI 0.84 to 2.87), respectively. Adjustment for maternal and infant baseline characteristics and covariates did not significantly alter the association between maternal smoking and infant development. Table 2 shows the unadjusted ORs with the different measures of cigarette smoke exposure, as well as the ORs adjusted by maternal and infant baseline characteristics.

**Table 2:** Multivariate models of the association between maternal tobacco smoke exposure in pregnancy and child developmental impairment at 2 years (analyses restricted to 884 mothers with measures of infant 2 year developmental outcome)

	ORs (95% CI) for developmental impairment at age 2	
	Unadjusted	Adjusted*
Daily number of cigarettes smoked at study randomisation	1.01 (0.98 to 1.04)	1.00 (0.97 to 1.03)
Maternal heaviness of smoking index at study randomisation (high vs low)	1.30 (0.87 to 1.95)	1.26 (0.84 to 1.90)
Maternal smoking cessation in pregnancy (measured 1 month after randomisation)	0.99 (0.59 to 1.66)	1.03 (0.61 to 1.75)
Maternal abstinence from smoking during latter stages of pregnancy (y/n)	1.55 (0.84 to 2.87)	1.53 (0.82 to 2.87)

*Adjusted for maternal age, gestational age at birth and infant birth weight.

## Discussion

### Summary of principal findings

We found no associations between maternal smoking status at different points during pregnancy and infants’ developmental impairment at 2 years. Consequently, there is no evidence to support the hypothesis that the better infant development observed within infants born to women who were randomised to NRT in the SNAP trial resulted from smoking cessation induced by nicotine patch use.

### Strengths and limitations

Data for this study are from the largest randomised controlled trial of NRT in pregnancy in which participants, healthcare and research staff were masked to treatment allocation.^14^ Follow-up and outcome ascertainment rates were high and there were only minor differences in characteristics of participants lost to follow-up and those remaining in the study at 2 years.^15^ It remains the only trial of a smoking cessation intervention in pregnancy to monitor infant outcomes; as such it is the only source of data which could be used to investigate the study hypothesis.

The ASQ-3 is a well-validated screening tool which has a sensitivity of 100% and specificity of 93% for detecting severe developmental delay at 24 months^20^. While some study infants may have been falsely identified as developmentally impaired, there is no reason to believe that such ASQ-3 false-positives would have occurred more frequently in infants of mothers who smoked in pregnancy. While developmental impairment was assessed in some infants using the participant questionnaire, others had the health professional questionnaire as the main source of data on impairment. Although this consequently implies a non-standardised assessment of the outcome in all infants, findings from the main study had shown that the proportion of participants assessed with either the PQ2 or the HPQ were similar in the treatment and placebo arms. Also, there were similarities in the baseline characteristics and birth outcomes of participants who returned the PQ2 and those for whom the HPQs were completed.

Although we adjusted for some potentially confounding factors which are known to be associated with infant development such as maternal socioeconomic status,^21^ maternal education,^22^ low birth weight,^23^ maternal exposure to passive smoking^24^ and maternal obesity,^25^ we were unable to adjust for those on which we had no data, such as maternal nutrition,^26^ depressive illness,^27^ stress and anxiety^28^ and alcohol consumption^29^ which could also potentially influence infant development but which were not measured in the SNAP trial. There remains therefore, the potential for residual confounding.

We undertook secondary analyses of randomised controlled trial data and the original sample size was intended to have sufficient power only for detecting differences in validated smoking cessation rates at the end of pregnancy. Analyses presented here may not have sufficient power to demonstrate associations between smoking and infant development; however, one would expect to find one or more such associations if, the relationship between NRT use and infant development which was demonstrated within the same data, were principally explained by smoking cessation caused by NRT.

There may have been some misattribution of smoking status. Measures of smoking status used were biochemically-validated at two different time points in pregnancy. Validation with carbon monoxide at the earlier time point could only eliminate smoking within the previous 24 hours^30^; and at delivery, with saliva could only do so for the previous 7 days.^17^ Consequently, some participants may have relapsed or smoked occasionally between these times^31^; however we believe it is reasonable to assume that women who were validated as not smoking both at the end of pregnancy and at 1 month after randomisation would have lower overall tobacco smoke exposure than those who were not. As recruitment into the study occurred between 12 and 24 weeks gestation, the timing of smoking status measurements in relation to gestation will have differed between participants but there is no evidence that smoking is more or less harmful at any point in pregnancy. Although these were the best available data for analyses, the assumptions outlined above need to be considered when interpreting study findings.

As most trial participants continued to smoke, analyses might have been more illuminating if data on the intensity of women’s smoking during pregnancy had been available. For example, if exhaled CO measurements or numbers of cigarettes smoked daily in pregnancy had been available for non-abstinent trial participants, these data could have been used to investigate whether smoking intensity in pregnancy was associated infant development. Increasing smoking intensity, reflected by exhaled CO levels, is associated with reduced infant birth weights,^32^ so an association with development seems possible.

Our analyses did not incorporate postnatal environmental cigarette smoke exposure which may have an independent influence on infant development.^33^ We know that there is continued maturation of the connections between brain regions after birth, with intense brain white matter myelination in the first postnatal months which becomes progressively less rapid through toddlerhood until young adulthood.^34^ Additionally, postnatal exposure to environmental tobacco is associated with emotional and conduct behavioural problems in school aged children^35^ and so may have an aetiological role in infant developmental impairment. As maternal prenatal and postnatal smoking are strongly correlated, this would increase the likelihood of finding associations between smoking in pregnancy and infant development; as none were found there is no suggestion of a large impact on findings. Nonetheless, there is a need to account for environmental cigarette smoke exposure in future studies.

### Comparison with literature

As this is an original analysis in a unique trial database there is very little literature with which study findings can be compared. Previous observational studies have found prenatal tobacco exposure to be associated with offspring adverse consequences including behavioural^36^ and hyperactivity^37 38^ problems, language and reading deficits,^37^ conduct disorders,^39^ newborn basic perceptual skills^40^ and intellectual impairment in childhood^41^ and later life.^42^ However, other studies which adjusted for potentially confounding factors such as maternal education^43 44^ and parental socioeconomic status^45^ have not. Consistent with these latter studies, our findings found no evidence that the improved developmental outcomes in infants of pregnant smokers who used NRT in the SNAP trial^15^ were explained by smoking cessation. As preclinical studies show that nicotine is neurotoxic and can adversely affect the developing central nervous system,^11 12^ it seems unlikely that these better outcomes are due to a direct protective effect of NRT on the developing foetus. Also, compliance rates in the NRT patch and placebo study arms were low in the SNAP trial (7.2% and 2.8% compliance, respectively, at 1 month). Perhaps, it is possible that nicotine used in pregnancy has no impact on infants’ developmental outcomes and the apparently protective effect of NRT on developmental outcomes observed in the SNAP trial occurred by chance.

## Conclusion

We found no evidence that the better 2 year developmental outcomes in offspring of pregnant women randomised to NRT patch use in the SNAP trial was due to the smoking cessation caused by NRT. Future research which takes account of women’s intensity of smoking in pregnancy as well as important maternal confounders and postnatal exposures, is needed to investigate how NRT use in pregnancy may exert this effect on infant development.

## Supplementary Material

Reviewer comments

Author's manuscript

## References

[1] Smoking and the Young. A report of a working party of the Royal College of Physicians. London: RCP, 1992.

[2] Mohsin M, Bauman AE Socio-demographic factors associated with smoking and smoking cessation among 426,344 pregnant women in New South Wales, Australia. BMC Public Health 2005;5:138 10.1186/1471-2458-5-138 16371166 PMC1352372

[3] Egebjerg Jensen K, Jensen A, Nøhr B, et al Do pregnant women still smoke? A study of smoking patterns among 261,029 primiparous women in Denmark 1997-2005. Acta Obstet Gynecol Scand 2008;87:760–7. 10.1080/00016340802179814 18696277

[4] Centers for Disease Control and Prevention (CDC). Smoking during pregnancy-United States, 1990-2002. MMWR Morb Mortal Wkly Rep 2004;53:4. 15470322

[5] McAndrew F, Thompson J, Fellows L, et al Infant Feeding Survey 2010: Health and Social Care Information Centre. 2012.

[6] Oncken CA, Dietz PM, Tong VT, et al Prenatal tobacco prevention and cessation interventions for women in low- and middle-income countries. Acta Obstet Gynecol Scand 2010;89:442–53. 10.3109/00016341003678450 20235895 PMC3918940

[7] World Health Organization. Report on the Global Tobacco Epidemic 2008—the mpower package. Geneva: World Health Organization, 2008.

[8] Chamberlain C, O’Mara-Eves A, Oliver S, et al Psychosocial interventions for supporting women to stop smoking in pregnancy. Cochrane Database Syst Rev 2013;10:CD001055 10.1002/14651858.CD001055.pub4 PMC4022453 24154953

[9] Stead LF, Perera R, Bullen C, et al Nicotine replacement therapy for smoking cessation. Cochrane Database Syst Rev 2012;11:CD000146 10.1002/14651858.CD000146.pub4 23152200

[10] Dhalwani NN, Szatkowski L, Coleman T, et al Prescribing of nicotine replacement therapy in and around pregnancy: a population-based study using primary care data. Br J Gen Pract 2014;64:e554–60. 10.3399/bjgp14X681361 25179069 PMC4141612

[11] Dempsey DA, Benowitz NL Risks and benefits of nicotine to aid smoking cessation in pregnancy. Drug Saf 2001;24:277–322. 10.2165/00002018-200124040-00005 11330657

[12] Benowitz N, Dempsey D Pharmacotherapy for smoking cessation during pregnancy. Nicotine Tob Res 2004;6(Suppl 2):S189–202. 10.1080/14622200410001669169 15203821

[13] Benowitz NL, Dempsey DA, Goldenberg RL, et al The use of pharmacotherapies for smoking cessation during pregnancy. Tob Control 2000;9(Suppl 3):III91–4. 10.1136/tc.9.suppl_3.iii91 10982920 PMC1766294

[14] Coleman T, Cooper S, Thornton JG, et al A randomized trial of nicotine-replacement therapy patches in pregnancy. N Engl J Med 2012;366:808–18. 10.1056/NEJMoa1109582 22375972

[15] Cooper S, Taggar J, Lewis S, et al Effect of nicotine patches in pregnancy on infant and maternal outcomes at 2 years: follow-up from the randomised, double-blind, placebo-controlled SNAP trial. Lancet Respir Med 2014;2:728–37. 10.1016/S2213-2600(14)70157-2 25127405

[16] Heatherton TF, Kozlowski LT, Frecker RC, et al Measuring the heaviness of smoking: using self-reported time to the first cigarette of the day and number of cigarettes smoked per day. Br J Addict 1989;84:791–9. 2758152 10.1111/j.1360-0443.1989.tb03059.x

[17] Montalto NJ, Wells WO Validation of self-reported smoking status using saliva cotinine: a rapid semiquantitative dipstick method. Cancer Epidemiol Biomarkers Prev 2007;16:1858-62 10.1158/1055-9965.EPI-07-0189 17855706

[18] Protocol for the SNAP (Smoking Nicotine and Pregnancy) Trial Final version 70. 2009.

[19] Lee PH Is a cutoff of 10% appropriate for the change-in-estimate criterion of confounder identification? J Epidemiol 2014;24:161–7. 10.2188/jea.je20130062 24317343 PMC3983286

[20] Gollenberg AL, Lynch CD, Jackson LW, et al Concurrent validity of the parent-completed Ages and Stages Questionnaires, 2nd Ed. with the Bayley Scales of Infant Development II in a low-risk sample. Child Care Health Dev 2010;36:485–90. 10.1111/j.1365-2214.2009.01041.x 20030657

[21] Ronfani L, Vecchi Brumatti L, Mariuz M, et al The Complex Interaction between Home Environment, Socioeconomic Status, Maternal IQ and Early Child Neurocognitive Development: A Multivariate Analysis of Data Collected in a Newborn Cohort Study. PLoS One 2015;10:e0127052 10.1371/journal.pone.0127052 25996934 PMC4440732

[22] Carneiro P, Meghir C, Parey M Maternal education, home environments, and the development of children and adolescents. Journal of the European Economic Association 2013;11:123–60. 10.1111/j.1542-4774.2012.01096.x

[23] Stein RE, Siegel MJ, Bauman LJ Are children of moderately low birth weight at increased risk for poor health? A new look at an old question. Pediatrics 2006;118:217–23. 10.1542/peds.2005-2836 16818568

[24] Jorge JG, Botelho C, Silva AM, et al Influence of passive smoking on learning in elementary school. J Pediatr 2016;92:260–7. 10.1016/j.jped.2015.08.010 26859248

[25] Wylie A, Sundaram R, Kus C, et al Maternal prepregnancy obesity and achievement of infant motor developmental milestones in the upstate KIDS study. Obesity 2015;23:907–13. 10.1002/oby.21040 25755075 PMC4380825

[26] Marques AH, O’Connor TG, Roth C, et al The influence of maternal prenatal and early childhood nutrition and maternal prenatal stress on offspring immune system development and neurodevelopmental disorders. Front Neurosci 2013;7 10.3389/fnins.2013.00120 PMC3728489 23914151

[27] Ibanez G, Bernard JY, Rondet C, et al Effects of Antenatal Maternal Depression and Anxiety on Children’s Early Cognitive Development: A Prospective Cohort Study. PLoS One 2015;10:e0135849 10.1371/journal.pone.0135849 26317609 PMC4552796

[28] Tarabulsy GM, Pearson J, Vaillancourt-Morel MP, et al Meta-analytic findings of the relation between maternal prenatal stress and anxiety and child cognitive outcome. J Dev Behav Pediatr 2014;35:38–43. 10.1097/DBP.0000000000000003 24345757

[29] Knudsen AK, Skogen JC, Ystrom E, et al Maternal pre-pregnancy risk drinking and toddler behavior problems: the Norwegian Mother and Child Cohort Study. Eur Child Adolesc Psychiatry 2014;23:901–11. 10.1007/s00787-014-0588-x 25053124 PMC4186966

[30] Secker-Walker RH, Vacek PM, Flynn BS, et al Exhaled carbon monoxide and urinary cotinine as measures of smoking in pregnancy. Addict Behav 1997;22:671–84. 10.1016/S0306-4603(97)00013-0 9347069

[31] Parazzini F, Davoli E, Rabaiotti M, et al Validity of self-reported smoking habits in pregnancy: a saliva cotinine analysis. Acta Obstet Gynecol Scand 1996;75:352–4. 10.3109/00016349609033330 8638455

[32] Reynolds CME, Egan B, Kennedy RA, et al The implications of high carbon monoxide levels in early pregnancy for neonatal outcomes. Eur J Obstet Gynecol Reprod Biol 2019;233:6–11. 10.1016/j.ejogrb.2018.11.020 30529257

[33] Yolton K, Dietrich K, Auinger P, et al Exposure to environmental tobacco smoke and cognitive abilities among U.S. children and adolescents. Environ Health Perspect 2005;113:98–103. 10.1289/ehp.7210 15626655 PMC1253717

[34] Dubois J, Dehaene-Lambertz G, Kulikova S, et al The early development of brain white matter: a review of imaging studies in fetuses, newborns and infants. Neuroscience 2014;276:48–71. 10.1016/j.neuroscience.2013.12.044 24378955

[35] Chastang J, Baïz N, Cadwallader JS, et al Postnatal Environmental Tobacco Smoke Exposure Related to Behavioral Problems in Children. PLoS One 2015;10 10.1371/journal.pone.0133604 PMC4526529 26244898

[36] Carter S, Paterson J, Gao W, et al Maternal smoking during pregnancy and behaviour problems in a birth cohort of 2-year-old Pacific children in New Zealand. Early Hum Dev 2008;84:59–66. 10.1016/j.earlhumdev.2007.03.009 17499944

[37] Naeye RL, Peters EC Mental development of children whose mothers smoked during pregnancy. Obstet Gynecol 1984;64:601–7. 6493652

[38] Langley K, Holmans PA, van den Bree MB, et al Effects of low birth weight, maternal smoking in pregnancy and social class on the phenotypic manifestation of Attention Deficit Hyperactivity Disorder and associated antisocial behaviour: investigation in a clinical sample. BMC Psychiatry 2007;7:26 10.1186/1471-244X-7-26 17584500 PMC1913513

[39] Wakschlag LS, Lahey BB, Loeber R, et al Maternal smoking during pregnancy and the risk of conduct disorder in boys. Arch Gen Psychiatry 1997;54:670–6. 10.1001/archpsyc.1997.01830190098010 9236551

[40] Key AP, Ferguson M, Molfese DL, et al Smoking during pregnancy affects speech-processing ability in newborn infants. Environ Health Perspect 2007;115:623–9. 10.1289/ehp.9521 17450234 PMC1852679

[41] Olds DL, Henderson CR, Tatelbaum R Intellectual impairment in children of women who smoke cigarettes during pregnancy. Pediatrics 1994;93:221–7. 8121734

[42] Mortensen EL, Michaelsen KF, Sanders SA, et al A dose-response relationship between maternal smoking during late pregnancy and adult intelligence in male offspring. Paediatr Perinat Epidemiol 2005;19:4–11. 10.1111/j.1365-3016.2004.00622.x 15670102

[43] Batty GD, Der G, Deary IJ Effect of maternal smoking during pregnancy on offspring’s cognitive ability: empirical evidence for complete confounding in the US national longitudinal survey of youth. Pediatrics 2006;118:943–50. 10.1542/peds.2006-0168 16950984

[44] Breslau N, Paneth N, Lucia VC, et al Maternal smoking during pregnancy and offspring IQ. Int J Epidemiol 2005;34:1047–53. 10.1093/ije/dyi163 16085682

[45] Roza SJ, Verhulst FC, Jaddoe VW, et al Maternal smoking during pregnancy and child behaviour problems: the Generation R Study. Int J Epidemiol 2009;38:680–9. 10.1093/ije/dyn163 18775874

