# Supplementary material for: Is the apparently protective effect of maternal nicotine replacement therapy (NRT) used in pregnancy on infant development explained by smoking cessation?: secondary analyses of a randomised controlled trial
Source: BMJ Open. 2019 Jul 11;9(7):e024923. doi: 10.1136/bmjopen-2018-024923 (PMC6629395; doi:10.1136/bmjopen-2018-024923)
Supplement: Author's manuscript [file bmjopen-2018-024923.draft_revisions.pdf]

BMJ Open

BMJ Open is committed to open peer review. As part of this commitment we make the peer review history of every article we publish publicly available.

When an article is published we post the peer reviewers' comments and the authors' responses online. We also post the versions of the paper that were used during peer review. These are the versions that the peer review comments apply to.

The versions of the paper that follow are the versions that were submitted during the peer review process. They are not the versions of record or the final published versions. They should not be cited or distributed as the published version of this manuscript.

BMJ Open is an open access journal and the full, final, typeset and author-corrected version of record of the manuscript is available on our site with no access controls, subscription charges or pay-per-view fees (http://bmjopen.bmj.com).

If you have any questions on BMJ Open's open peer review process please email info.bmjopen@bmj.com

BMJ Open

Is the apparently protective effect of maternal nicotine replacement therapy (NRT) used in pregnancy on infant development explained by smoking cessation?: secondary analyses of a randomised controlled trial

Journal:	*BMJ Open*
Manuscript ID	bmjopen-2018-024923
Article Type:	Research
Date Submitted by the Author:	21-Jun-2018

Complete List of Authors:	Iyen, Barbara; University of Nottingham, Division of Primary Care<br>Vaz, Luis; University of Nottingham Division of Primary Care,<br>Taggar, Jaspal; University of Nottingham Faculty of Medicine and Health Sciences, Division of Primary Care, School of Medicine<br>Cooper, Sue; University of Nottingham, Division of Primary Care<br>Lewis, Sarah; University of Nottingham, Division of Epidemiology and Public Health<br>Coleman, Tim; University of Nottingham, Division of Primary Care

Keywords:	Maternal medicine < OBSTETRICS, Developmental neurology & neurodisability < PAEDIATRICS, EPIDEMIOLOGY

SCHOLARONE™ Manuscripts

For peer review only

Is the apparently protective effect of maternal nicotine replacement therapy (NRT) used in pregnancy on infant development explained by smoking cessation?: secondary analyses of a randomised controlled trial

Barbara Iyen[1], PhD., Luis R. Vaz[1], PhD., Jaspal Taggar[1], PhD., Sue Cooper[1], PhD., Sarah Lewis[2], PhD., Tim Coleman[1],MD.

Affiliations: [1]Division of Primary care, University of Nottingham, UK and [2]Division of Epidemiology and Public health, University of Nottingham, UK

Address correspondence to:
Barbara Iyen
Division of Primary Care
University of Nottingham,
Tower Building, Room 1402, Nottingham
Email: Barbara.iyen@nottingham.ac.uk;
Tel.: +44 115 84 66588
Fax: +44 115 84 66904

Short title:
Maternal exposure to tobacco smoke in pregnancy and infant development at age 2

Keywords:
Smoking in pregnancy, maternal smoking, child development, developmental impairment

Word count (abstract): 275
Word count (text): 3,150

For peer review only - http://bmjopen.bmj.com/site/about/guidelines.xhtml

ABSTRACT

Objective: To investigate relationships between maternal smoking status in pregnancy and infant development. The largest RCT of nicotine replacement therapy (NRT) for smoking cessation in pregnancy, the Smoking and Nicotine in Pregnancy (SNAP) trial, found that infants born to women randomised to NRT were more likely to have unimpaired development at 2 years. We hypothesized that this apparently protective effect was due to smoking cessation caused by NRT and investigate this relationship using the same cohort

Design: Secondary analysis of a randomised controlled trial

Setting: Seven antenatal hospitals in the Midlands and North-West England

Participants: One thousand and fifty women between 12 and 24 weeks gestation who smoked at least ten cigarettes/day before pregnancy and at least five cigarettes/day during pregnancy, were randomised to receive NRT patches or visually-identical placebo. Participants' smoking behaviour measures were recorded at baseline, one month after their target quit date and at delivery.

Methods: Using logistic regression models, we investigated associations between participants' smoking measures and infant development at 2 years.

Main outcome measures: 2-year infant development

Results: Developmental impairment was reported for 12.7% of study 2 year olds. Maternal baseline heaviness of smoking (odds ratio [OR]: 1.26, 95% confidence interval [95% CI]: 0.82-1.96, p = 0.091), validated smoking abstinence recorded at one month after a quit date (OR: 1.02, 95% CI: 0.60-1.74, p = 0.914) and validated smoking abstinence recorded at one month after a quit date and at the end of pregnancy (OR: 1.52, 95% CI: 0.81-2.85, p = 0.795) were not independently associated with infant developmental impairment at 2 years.

Conclusions: We found no evidence that NRT improved infants' developmental outcomes through an effect on maternal smoking.

For peer review only - http://bmjopen.bmj.com/site/about/guidelines.xhtml

ARTICLE SUMMARY

Strengths and limitations of this study

- This study uses data from the smoking and nicotine in pregnancy (SNAP) trial, the largest randomised controlled trial of nicotine replacement therapy (NRT) for smoking cessation in Pregnancy.
- The SNAP trial remains the only maternal smoking cessation trial to investigate infant outcomes beyond delivery.
- Baseline characteristics and potential confounders were included in multivariable analyses to determine independent associations between maternal smoking and 2-year developmental outcomes.
- Maternal factors which were not measured in the SNAP trial could not be adjusted for in multivariable analyses.

For peer review only

For peer review only - http://bmjopen.bmj.com/site/about/guidelines.xhtml

INTRODUCTION

Smoking in pregnancy is associated with increased risks of many adverse outcomes including miscarriage, stillbirth, prematurity, low birth weight, perinatal morbidity and mortality[1] and is a substantial international public health problem; in high income countries 13% to 25% of pregnant women smoke [2-5] and in developing countries[6 7], the World Health Organisation predicts a future epidemic. Fortunately, impacts on the fetus are avoidable and cessation in pregnancy improves infants' birth weights and reduces the risk of premature birth.[8] Additionally, pregnancy is the life event which seems to most motivate smokers' cessation attempts; over 50% of UK pregnant smokers try stopping.[5] Women are, therefore, likely to be receptive to support with stopping and, as it is effective for non-pregnant smokers[9] and possibly also for pregnant smokers too, nicotine replacement therapy (NRT) is frequently offered to pregnant women who smoke[10]. Although NRT is not thought to be completely risk-free because it exposes users to nicotine, this is considered safer than smoking because smokers are already nicotine-exposed and NRT users are not exposed to all the numerous tobacco smoke carcinogens.

Animal experiments and human-subject laboratory investigations suggest that nicotine may adversely affect the fetal cardiovascular system and developing nerve tissue[11 12], however trials of NRT for smoking cessation in pregnancy have been permitted because any fetal harm from the nicotine would likely be compensated for by the benefits of mothers stopping smoking[13]. In the largest of these trials, nicotine patches doubled cessation rates at four weeks but by delivery there was no difference in cessation rate between the NRT and placebo groups.[14] Unexpectedly, infants born to NRT group women were 8% more likely to have unimpaired development at two years of age than infants born to women in the placebo groups[15] and there was a dose-response relationship between adherence to NRT and impairment-free infant development.[15] It seems unlikely that this effect would be due to nicotine having a direct beneficial effect on the fetus. As there was a substantial impact on cessation in early pregnancy, we hypothesise that the better developmental

For peer review only - http://bmjopen.bmj.com/site/about/guidelines.xhtml

outcomes in NRT group infants and the dose-response relationship between increasing NRT use and these might be explained by smoking cessation during pregnancy caused by NRT use. Consequently, we present secondary analyses of data from the same trial to investigate whether or not the absence of infants' developmental impairments at two years was associated with maternal smoking status measured at different points in the trial.

METHODS

Study participants

We used data from the Smoking, Nicotine and Pregnancy (SNAP) trial, a placebo-randomised -controlled trial of NRT for smoking cessation in pregnant women[14]. The trial recruited 1,050 participants aged 16-45 years between 12 and 24 weeks gestation who smoked at least ten cigarettes a day before pregnancy and at least five cigarettes a day during pregnancy, with exhaled carbon monoxide (CO) readings of ≥8ppm. Participants were randomised to receive up to an 8-week course of either 15mg per 16 hours of NRT patches or visually-identical placebo. For safety reasons, women were instructed that if they smoked at all whilst using patches, the NRT should be stopped. Participants set a target quit date and were followed up one month after this, during hospital admission for delivery or as soon as possible afterwards, and at 24 months after delivery.

Maternal baseline data and smoking behaviour measures

Baseline data included date of birth, age on leaving full time education, number of cigarettes smoked per day prior to and during pregnancy, body mass index (BMI), the heaviness of smoking index (HSI) - a measure of nicotine dependence derived from the time to first cigarette after waking and the number of cigarettes smoked daily,[16] previous use of NRT in the current pregnancy and partners' smoking status .

For peer review only - http://bmjopen.bmj.com/site/about/guidelines.xhtml

For peer review only

Self-reported smoking data were obtained one month after the target quit date and at delivery. Self-reported smoking abstinence at one-month after the quit date was validated by exhaled carbon monoxide (CO) measurements below 8ppm while abstinence at delivery was validated by CO measurements and estimated saliva cotinine (COT) concentrations below 10ng/ml. No data on tobacco smoke exposure were collected between these two time-points. Self-reported data on the use of nicotine patches were requested one month after the target quit date and at delivery; adherence to NRT have been reported elsewhere[14].

Infant outcome measures

Birth status (live or still birth), birth weight, singleton or multiple birth, sex and gestational age at birth were recorded at delivery and have been reported elsewhere[14]. The presence of infant developmental impairment at 24 months was determined using questionnaire responses from participants or from healthcare professionals when there was no participant response; full details have been described previously[15]. Participant questionnaires were posted out at 24 months and if no response was received, health professional ones were dispatched to non-respondents' family physicians. Participants reported on their infants' development as assessed using the 5 domains of the Ages and Stages Questionnaire edition 3 (ASQ-3): communication, gross motor, fine motor, problem solving and personal-social skills.[15] Scores from the five ASQ-3 domains and responses to 'non-domain' ASQ-3 items in the participant questionnaires were used with established norms to categorise infants as being 'impaired' or 'not impaired'. Health professional questionnaire (HPQ) items were consistent with domains in participant questionnaires and were designed to be completed with reference to medical or health visitor records. Infants with only HPQ responses were considered to have no developmental impairment when the responses from all questions indicated no potential developmental issues.[15]

For peer review only - http://bmjopen.bmj.com/site/about/guidelines.xhtml

Measures of tobacco smoke exposure

As outlined above, smoking behaviour measures were collected at baseline, one month after a target quit date and at the end of pregnancy. From these data, we derived the following categories to represent different levels of tobacco smoke exposure during pregnancy:

i) Baseline number of cigarettes smoked daily during pregnancy

ii) Baseline heaviness of smoking index (HSI) during pregnancy, categorised as high (4-6) or low (0-3)

iii) Women who reported not smoking between their quit date and one month, validated using exhaled CO.

iv) Women who reported not smoking between their target quit date and the end of pregnancy or shortly afterwards, validated by either or both of exhaled CO and saliva cotinine at the end of pregnancy and also by exhaled CO at one month after their quit date.

As all trial participants smoked at baseline, exposure category (ii) was intended to dichotomise those who smoked more heavily and less heavily in the first weeks of pregnancy. For categories (iii) and (iv), we hypothesised that those demonstrating validated abstinence at both one month and the end of pregnancy (category iv) would have a lower overall tobacco smoke exposure than those for whom abstinence was only validated at one month after their quit date. However, it should be noted that no biochemical test can validate smoking cessation for a prolonged period and some women in either group may have smoked for a time between baseline / study enrolment and one month or the end of pregnancy.

For peer review only - http://bmjopen.bmj.com/site/about/guidelines.xhtml

Analyses

Analyses were carried out for participants who had live singleton births. Univariable logistic regression was used to investigate the association between 2-year developmental outcomes and different measures of maternal tobacco smoke exposure such as baseline number of cigarettes smoked daily in pregnancy, the heaviness of smoking index during pregnancy, maternal smoking abstinence at some point during pregnancy and maternal smoking abstinence during the latter stages of pregnancy. Multivariable logistic regression analyses were then conducted to determine the independent associations between the measures of maternal smoking exposure and infant development outcomes. We used a forward stepwise regression to identify potential confounder variables; we included baseline characteristic variables that were found to be significantly associated with maternal smoking exposures and developmental impairment and we also controlled for variables that are well known to be associated with childhood development. Potential confounders included in the multivariable analyses were: birth weight, maternal age, age at which full time education was completed, maternal BMI and partner's smoking status.

All analyses were performed using Stata 14. Significant associations were defined at the $p \leq 0.05$ significance level.

For peer review only

For peer review only - http://bmjopen.bmj.com/site/about/guidelines.xhtml

RESULTS

From 1,050 pregnant smokers enrolled in the SNAP trial there were 12 women with twin pregnancies, 14 participants who had fetal deaths, 4 lost to follow-up and 10 who withdrew consent. Analyses included 1,010 women with singleton live births (Figure 1). The self-reported median (IQR) number of daily cigarettes smoked by women prior to pregnancy was 19 (10-60). At the time of recruitment into the study, the reported median (IQR) number of cigarettes smoked daily in pregnancy was 15 (10-20). Of the infants born to participants in the study, 52% were males and less than 10% had low birth weight. Data on developmental impairment was available for 884 infants at age 2 and impairment was present in 12.7% of these children. (n=112).

Study participants and singleton infants with 2-year outcome data had similar baseline characteristics to participants reported in the full trial cohort[15]. Aside from the fact that participants who were lost to follow-up had higher mean IMD (Index of multiple deprivation) scores implying that they lived in more deprived areas than participants who had data up to 2 years, there were only minor differences in the baseline characteristics between the participants with 2-year data and those who were lost to follow-up[15].

Figure 1. Flow diagram of study participants

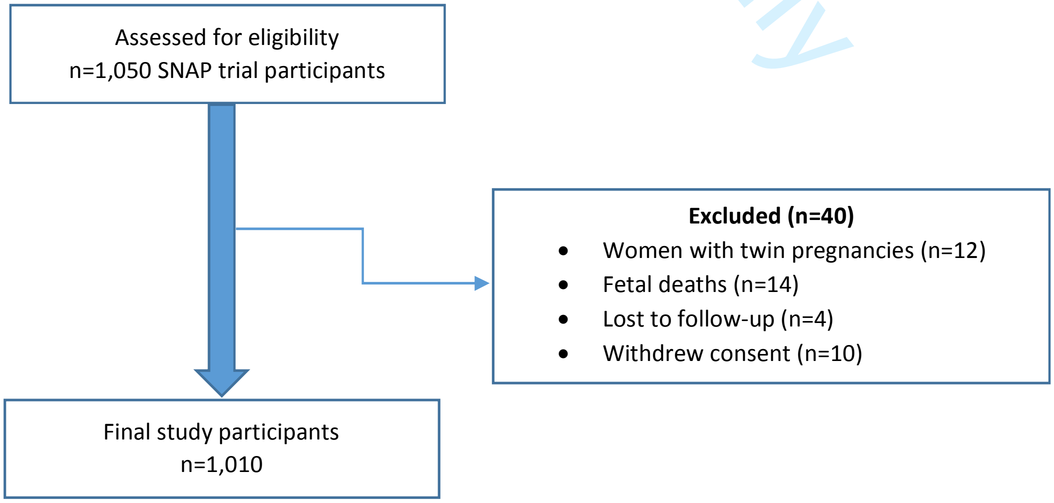

For peer review only - http://bmjopen.bmj.com/site/about/guidelines.xhtml

For peer review only

Maternal and infant characteristics

The characteristics of the women and children included in the analyses and their associations with infant developmental impairment at two years are presented in table 1.

The majority of participants included in the study (72.4%) were less than 30 years old, 39.9% had a BMI between 18.5-24.9kg/m^2. The average age of leaving full time education was 16.3 years. We used the heaviness of smoking index as a measure of the level of maternal nicotine dependence and 65% of women in the study had low levels of nicotine dependence prior to being recruited in the study. More than two-thirds of the study participants had partners who smoked (67.9%). Eighteen percent (18%) of women were validated as abstinent from smoking at some point during pregnancy (measured one month after their target quit date) and 8.9% remained abstinent at the latter stages of pregnancy until delivery. There was no statistically significant relationship between smoking exposure or abstinence and infant developmental impairment. The only variable that was found to be significantly associated with developmental impairment in univariable analysis was infant sex which was retained in addition to the a priori defined variables, as a confounder in multivariable analyses.

For peer review only

For peer review only - http://bmjopen.bmj.com/site/about/guidelines.xhtml

Table 1. Maternal and infant characteristics with univariable association between these and infant developmental impairment (N=884)

	n (%)	Unadjusted OR (95% CI) for developmental impairment	p-value
**Maternal characteristics**			
Maternal age (years) at pregnancy (n=884)			
<20	136 (15.4%)	1.00	0.501
20-24	292 (33.0%)	0.62 (0.34-1.11)	
25-29	212 (24.0%)	0.63 (0.34-1.18)	
30-34	148 (16.7%)	0.95 (0.50-1.80)	
35-39	74 (8.4%)	0.81 (0.36-1.82)	
>40	22 (2.5%)	0.82 (0.22-3.00)	
Maternal BMI* [kg/m^2] (n=847)			
<18.5	23 (2.7%)	0.57 (0.15-2.89)	0.853
18.5-24.9	338 (39.9%)	1.00	
25.0-29.9	252 (29.8%)	0.96 (0.59-1.58)	
>30.0	234(27.6%)	1.13 (0.69-1.83)	
Maternal age of leaving full time education** (n=872)	16.3 (1.5)	1.09 (0.98-1.21)	0.698
Partner's smoking status (n=884)			
Non smoker	214 (24.2%)	1.00	0.899
Smoker	600 (67.9%)	1.05 (0.65-1.69)	
No data on partner's smoking status	70 (7.9%)	1.21 (0.55-2.64)	
Heaviness of smoking index (HSI) during pregnancy (N=884)			
Low index (0-3)	576 (65.2%)	1.00	0.209
High index (4-6)	308 (34.8%)	1.30 (0.87-1.95)	
Daily number of cigarettes smoked during pregnancy^ (n=884)	15.0 (10-20)	1.01 (0.98-1.04)	0.674
Daily number of cigarettes smoked during pregnancy in categories (n=884)			
5-9/day	172 (19.5%)	1.00	0.29
10-19/day	482 (54.5%)	1.08 (0.62-1.87)	
>20/day	230 (26.0%)	1.49 (0.82-2.71)	
Maternal abstinence from smoking at some point during pregnancy (n=884)			
Did not abstain	725 (82.0%)	1.00	0.970
Abstained	159 (18.0%)	0.99 (0.59-1.66)	
Maternal abstinence from smoking during latter stages of pregnancy (n=884)			
Did not abstain	805 (91.1%)	1.00	0.176
Abstained	79 (8.9%)	1.55 (0.84-2.87)	
**Infant characteristics**			
Sex of baby (n=884)			
Female	422 (47.7%)	1.00	0.003
Male	462 (52.3%)	1.84 (1.22-2.79)	
Birth weight (n=884)			
<2.5kg	85 (9.6%)	1.00	0.408
2.5-3.0kg	215 (24.3%)	0.56 (0.28-1.13)	
3.0-3.5kg	335 (37.9%)	0.63 (0.33-1.21)	
>3.5kg	249 (28.2%)	0.74 (0.38-1.43)	

*Maternal BMI categories defined as underweight "<18.5", normal weight "18.5-24.9", overweight "25.0-29.9", obese ">30.0"

All values are represented as n (%) unless indicated by ** which denotes mean (SD) or ^ which denotes median (interquartile range)

For peer review only - http://bmjopen.bmj.com/site/about/guidelines.xhtml

Table 2. Multivariate models of the association between maternal tobacco smoke exposure in pregnancy and child developmental impairment at 2 years (analyses restricted to 884 mothers with measures of infant 2-year developmental outcome)

	Odds ratio (95% CI) for developmental impairment at age 2	P-value
**Daily number of cigarettes smoked during pregnancy**		
Unadjusted	1.01 (0.98-1.04)	0.674
Adjusted ▪	1.01 (0.98-1.05)	0.099
**Maternal heaviness of smoking index during pregnancy (high vs low)**		
Unadjusted	1.30 (0.87-1.95)	0.209
Adjusted ▪	1.26 (0.82-1.96)	0.091
**Maternal abstinence from smoking at some point during pregnancy**		
Unadjusted	0.99 (0.59-1.66)	0.970
Adjusted ▪	1.02 (0.60-1.74)	0.914
**Maternal abstinence from smoking during latter stages of pregnancy**		
Unadjusted	1.55 (0.84-2.87)	0.176
Adjusted ▪	1.52 (0.81-2.85)	0.795

▪ Adjusted for infant birthweight, infant sex, maternal age, age of leaving full time education, maternal BMI and partners' smoking status

The multivariable analyses of the associations between different measures of maternal tobacco smoke exposure and infant developmental impairment are presented in table 2. There remained no statistically significant associations between maternal heaviness of smoking or abstinence from tobacco smoke exposure and infant developmental impairment at two years. The odds ratio for child developmental impairment in infants of mothers who abstained from smoking at some point during pregnancy and in infants of mothers who abstained during the latter stages of pregnancy until delivery, were 0.99 (95% CI 0.59-1.66) and 1.55 (95% CI 0.84-2.87) respectively. On adjusting for measured co-variates and a priori variables, these estimates remained statistically non-significant with odds ratios of 1.02 (95% CI 0.60-1.74) and 1.52 (95% CI 0.81-2.85) respectively.

For peer review only - http://bmjopen.bmj.com/site/about/guidelines.xhtml

DISCUSSION

Summary of principal findings

We found no associations between maternal smoking status at different points during pregnancy and infants' developmental impairment at two years. Consequently, there is no evidence to support the hypothesis that the better infant development observed within infants born to women who were randomised to NRT in the Smoking, Nicotine and Pregnancy (SNAP) trial resulted from smoking cessation induced by nicotine patch use.

Strengths and limitations

Data for this study are from the largest randomised controlled trial of NRT in pregnancy in which participants, health care and research staff were masked to treatment allocation[14]. Follow-up and outcome ascertainment rates were high and there were only minor differences in characteristics of participants lost to follow up and those remaining in the study at 2 years[15]. It remains the only trial of a smoking cessation intervention in pregnancy to monitor infant outcomes; as such it is the only source of data which could be used to investigate the study hypothesis.

The ASQ-3 is a well-validated screening tool which has a sensitivity of 100% and specificity of 93% for detecting severe developmental delay at 24 months[17]. Some study infants may have been falsely identified as developmentally impaired, but there is no reason to believe that such ASQ-3 false positives would have occurred more frequently in infants of mothers who smoked in pregnancy.

Although we adjusted for some potentially confounding factors which are known to be associated with infant development such as maternal socioeconomic status[18], maternal education[19], low birth weight[20] and maternal exposure to passive smoking[21], we were unable to adjust for those on which we had no data, such as maternal pre-pregnancy obesity, which has been found to be associated with delayed infant developmental milestones[22]. Additionally, maternal factors like nutrition[23], depressive illness[24], stress and anxiety[25] and alcohol consumption[26] which could also potentially

For peer review only - http://bmjopen.bmj.com/site/about/guidelines.xhtml

influence infant development were not measured in the SNAP trial, so there remains the potential for residual confounding. However, no analyses had borderline significant results so such confounding would need to have substantial impact to mask associations and this seems unlikely.

A limitation is that we undertook secondary analyses of randomised controlled trial data and the original sample size was intended to have sufficient power only for detecting differences in validated smoking cessation rates at the end of pregnancy. Analyses presented here may not have sufficient power to demonstrate associations between smoking and infant development; however one would expect to find one or more such associations if, the relationship between NRT use and infant development which was demonstrated within the same data, were explained by smoking cessation caused by NRT.

There may have been some misattribution of smoking status. Measures of smoking status used were biochemically-validated at two different time points in pregnancy. Validation with carbon monoxide at the earlier time point could only eliminate smoking within the previous 24 hours, and at delivery, with saliva could only do so for the previous seven days. Consequently, some participants may have relapsed or smoked occasionally between these times[27]; however we believe it is reasonable to assume that women who were validated as not smoking both at the end of pregnancy and at one month after randomisation would have lower overall tobacco smoke exposure than those who were not. As recruitment into the study occurred between 12 and 24 weeks gestation, the timing of smoking status measurements in relation to gestation will have differed between participants but there is no evidence that smoking is more or less harmful at any point in pregnancy. Although these were the best available data for analyses, the assumptions outlined above need to be considered when interpreting study findings.

Our analyses did not incorporate postnatal environmental cigarette smoke exposure which may have an independent influence on infant development. We know that there is continued maturation of the connections between brain regions after birth, with intense brain white matter myelination in

For peer review only - http://bmjopen.bmj.com/site/about/guidelines.xhtml

the first post-natal months which becomes progressively less rapid through toddlerhood until young adulthood[28]. Additionally, postnatal exposure to environmental tobacco is associated with emotional and conduct behavioural problems in school aged children[29] and so may have an aetiological role in infant developmental impairment. As maternal prenatal and postnatal smoking are strongly correlated, this would increase the likelihood of finding associations between smoking in pregnancy and infant development; as none were found there is no suggestion of a large impact on findings.

Comparison with literature

As this is an original analysis in a unique trial database there is very little literature with which study findings can be compared. Previous observational studies have found prenatal tobacco exposure to be associated with offspring adverse consequences including behavioural[30] and hyperactivity[31 32] problems, language and reading deficits[31], conduct disorders[33], newborn basic perceptual skills[34] and intellectual impairment in childhood[35] and later life[36]. However, other studies which adjusted for potentially confounding factors such as maternal education [37 38] and parental socioeconomic status[39]have not. Consistent with these latter studies, our findings found no evidence that the improved developmental outcomes in infants of pregnant smokers who used NRT in the SNAP trial[15] were explained by smoking cessation. As pre-clinical studies show that nicotine is neurotoxic and can adversely affect the developing central nervous system[11 12], it also seems unlikely that these better outcomes might be explained by a protective effect of NRT on fetal development acting independently of any smoking-cessation in pregnancy. Although unlikely, this is still a potential explanation and a study with greater power might resolve this important question. Perhaps, it is possible that nicotine used in pregnancy has no impact on infants' developmental outcomes and the apparently protective effect of NRT on developmental outcomes observed in the SNAP trial occurred by chance.

The strong univariate association between male infants and developmental impairment was an interesting and unexpected finding which we simply cannot explain. The ASQ-3 is utilised as a

For peer review only - http://bmjopen.bmj.com/site/about/guidelines.xhtml

gender-neutral tool however certain questions in the tool have been perceived by health professionals and parents to be gender-oriented and so, thought to potentially elicit more positive responses about either boys or girls[40]. In its routine application as part of the two-year health and development review, the ASQ-3 has been shown to be valid in identifying infants at risk of developmental impairment irrespective of gender[17 41]. To assess if our 'gender-developmental impairment' association was due to a gender-specific impact of nicotine from maternal smoking, we performed analysis of this association adjusting for maternal tobacco exposure, but the association was unchanged and remained strong. The relationship between gender and infant development would need to be explored further, however we know that this is not likely to have influenced the earlier finding of better developmental outcomes among infants born to women randomised to the NRT arm of the SNAP trial, as the proportion of male infants born to participants randomised to the NRT or placebo groups were similar at 49% and 51% respectively.

CONCLUSION

We found no evidence that the apparently protective effect of NRT patch use in pregnancy on infant development two years after delivery as seen in the Smoking, Nicotine and Pregnancy (SNAP) trial, was due to the smoking cessation in pregnancy caused by NRT. Further research is needed to investigate how NRT use in pregnancy may exert this observed effect on infant development.

For peer review only - http://bmjopen.bmj.com/site/about/guidelines.xhtml

For peer review only

ACKNOWLEDGEMENTS

We gratefully acknowledge the SNAP trial team members. In addition to listed authors, the complete trial team includes: Investigators : Kim Watts, Jim Thornton, Jo Leonardi-Bee, John Britton, Paul Aveyard, Michael Coughtrie, Christine Godfrey, Clare Mannion and Neil Marlow. Research staff: Janet Brown, Yvette Davis, Anne Dickinson, Caroline Dixon, Fiona Holloway, Joanne Lakin, Jayne Platts, Farzana Rashid, Amanda Redford, Cara Taylor. Principal investigators (in recruiting centres): Jonathan Allsop, Simon Cunningham, Karen Glass, Vince Hall, Khaled Ismail, Margaret Ramsay. Midwife leads (in recruiting centres): Sheena Appleby. Denise Bailey, Linda Gustard, Emma Haworth, Grace Hopps, Amanda Lindley, Chris Kettle, Colleen Pearce, Dymphna Sexton-Bradshaw, Julia Savage, Sandra Smith, Sheila Taylor, Alison Witham. Primary Care Trust & NHS Stop Smoking Services' Staff : Barbara Brady, Michelle Battlemuch, Wendy Dudley, Rochelle Edwards, Lorraine Frith, Indu Hari, Catriona Holden, Linda Hoskyns, Paul Jackson, Giri Rajaratnam, Deborah Richardson, Lucy Wade, Maureen Whittaker. QMC Pharmacy: Bernie Cook, Sheila Hodgson (Lead pharmacist), Lisa Humphries, Bernie Sanders (Qualified Person). University of Nottingham Clinical Trials Unit: Dan Simpkins. University of Dundee: Sheila Sharp.

COMPETING INTERESTS

The authors have no conflicts of interest relevant to this study.

FUNDING

This work was supported by the National Institute for Health Research (NIHR) Collaboration for Leadership in Applied Health Research and Care (CLAHRC) East Midlands at NHS Nottingham City CCG. BI's clinical academic lectureship is fully funded by the NIHR. TC, LV, SC and, SL, and TC are members of the UK Centre for Tobacco and Alcohol Studies (UKCTAS). TC, LV, SC and JT are members of the NIHR School for Primary care Research. Professor Coleman is a National Institute for Health Research Senior Investigator. The views and opinions expressed are those of the authors and do not necessarily reflect those of the NIHR Health Technology Assessment (HTA) Programme, the National Health Service (NHS), the NIHR or the Department of Health.

For peer review only - http://bmjopen.bmj.com/site/about/guidelines.xhtml

DETAILS OF ETHICS APPROVAL

National research ethical approval for the SNAP trial was granted by Oxfordshire Research Ethics Committee A, and additional local approvals for each recruitment centre and Clinical Trial Authorisation (CTA) approval was from the MHRA (CTA number: 03057/0002/001-0001). All participants gave written informed consent, including access to medical records for maintaining contact and for the follow-up of their child's health status.

AUTHOR STATEMENT

BI had full access to all of the data in the study and takes responsibility for the integrity of the data and the accuracy of the analyses. BI also wrote the first draft of the manuscript. LV contributed to the initial stages of data organisation, study concept and design, and proofread and approved the full manuscript prior to submission. TC contributed to the study hypothesis and design, data interpretation and analyses, assisted with writing up the "introduction" section and proofread and approved the final manuscript prior to submission. JT, SL and SC contributed to the data interpretation and analyses of this study as well as revision of the manuscript prior to submission.

For peer review only

For peer review only - http://bmjopen.bmj.com/site/about/guidelines.xhtml

REFERENCES

1. Smoking and the young. A report of a working party of the Royal College of Physicians. London: RCP, 1992.
2. Mohsin M, Bauman AE. Socio-demographic factors associated with smoking and smoking cessation among 426,344 pregnant women in New South Wales, Australia. *Bmc Public Health* 2005;5 doi: Artn 138

Doi 10.1186/1471-2458-5-138
3. Jensen KE, Jensen A, Nohr B, et al. Do pregnant women still smoke? A study of smoking patterns among 261,029 primiparous women in Denmark 1997-2005. *Acta Obstetricia Et Gynecologica Scandinavica* 2008;87(7):760-67. doi: Doi 10.1080/00016340802179814
4. Centers for Disease Control and Prevention. Smoking during pregnancy--United States, 1990-2002. *MMWR Morb Mortal Wkly Rep* 2004;39(53):4.
5. McAndrew F, Thompson J, Fellows L, et al. Infant Feeding Survey 2010: Health and Social Care Information Centre, 2012.
6. Oncken CA, Dietz PM, Tong VT, et al. Prenatal tobacco prevention and cessation interventions for women in low- and middle-income countries. *Acta Obstet Gynecol Scand* 2010;89(4):442-53. doi: 10.3109/00016341003678450 [published Online First: 2010/03/20]
7. World Health Organization. Report on the Global Tobacco Epidemic 2008—the mpower package. Geneva: World Health Organization, 2008.
8. Chamberlain C, O'Mara-Eves A, Oliver S, et al. Psychosocial interventions for supporting women to stop smoking in pregnancy. *Cochrane Database Syst Rev* 2013;10:CD001055. doi: 10.1002/14651858.CD001055.pub4
9. Stead LF, Perera R, Bullen C, et al. Nicotine replacement therapy for smoking cessation. *Cochrane Database Syst Rev* 2012;11:CD000146. doi: 10.1002/14651858.CD000146.pub4
10. Dhalwani NN, Szatkowski L, Coleman T, et al. Prescribing of nicotine replacement therapy in and around pregnancy: a population-based study using primary care data. *Br J Gen Pract* 2014;64(626):e554-60. doi: 10.3399/bjgp14X681361
11. Dempsey DA, Benowitz NL. Risks and benefits of nicotine to aid smoking cessation in pregnancy. [Review] [340 refs]. *Drug Safety* 2001;24(4):277-322.
12. Benowitz N, Dempsey D. Pharmacotherapy for smoking cessation during pregnancy. *Nicotine & Tobacco Research* 2004;6 Suppl 2:S189-202. doi: 10.1080/14622200410001669169 [published Online First: 2004/06/19]
13. Benowitz NL, Dempsey DA, Goldenberg RL, et al. The use of pharmacotherapies for smoking cessation during pregnancy. *Tobacco Control* 2000;9 Suppl 3:III91-III94.
14. Coleman T, Cooper S, Thornton JG, et al. A randomized trial of nicotine-replacement therapy patches in pregnancy. *New England Journal of Medicine* 2012;366(9):808-18. doi: http://dx.doi.org/10.1056/NEJMoa1109582
15. Cooper S, Taggar J, Lewis S, et al. Effect of nicotine patches in pregnancy on infant and maternal outcomes at 2 years: follow-up from the randomised, double-blind, placebo-controlled SNAP trial. *The Lancet Respiratory Medicine* 2014;2(9):728-37. doi: http://dx.doi.org/10.1016/S2213-2600(14)70157-2
16. Heatherton TF, Kozlowski LT, Frecker RC, et al. Measuring the heaviness of smoking: using self-reported time to the first cigarette of the day and number of cigarettes smoked per day. *British Journal of Addiction* 1989;84(7):791-99.
17. Gollenberg A, Lynch C, Jackson L, et al. Concurrent validity of the parent-completed Ages and Stages Questionnaires, 2nd Ed. with the Bayley Scales of Infant Development II in a low-risk sample. *Child Care Health and Development* 2010;36(4):485-90. doi: 10.1111/j.1365-2214.2009.01041.x

For peer review only - http://bmjopen.bmj.com/site/about/guidelines.xhtml

18. Ronfani L, Brumatti L, Mariuz M, et al. The Complex Interaction between Home Environment, Socioeconomic Status, Maternal IQ and Early Child Neurocognitive Development: A Multivariate Analysis of Data Collected in a Newborn Cohort Study. *Plos One* 2015;10(5) doi: 10.1371/journal.pone.0127052
19. Carneiro P, Meghir C, Parey M. MATERNAL EDUCATION, HOME ENVIRONMENTS, AND THE DEVELOPMENT OF CHILDREN AND ADOLESCENTS. *Journal of the European Economic Association* 2013;11:123-60. doi: 10.1111/j.1542-4774.2012.01096.x
20. Stein R, Siegel M, Bauman L. Are children of moderately low birth weight at increased risk for poor health? A new look at an old question. *Pediatrics* 2006;118(1):217-23. doi: 10.1542/peds.2005-2836
21. Jorge JG, Botelho C, Silva AM, et al. Influence of passive smoking on learning in elementary school. *J Pediatr (Rio J)* 2016;92(3):260-7. doi: 10.1016/j.jped.2015.08.010
22. Wylie A, Sundaram R, Kus C, et al. Maternal Prepregnancy Obesity and Achievement of Infant Motor Developmental Milestones in the Upstate KIDS Study. *Obesity* 2015;23(4):907-13. doi: 10.1002/oby.21040
23. Marques A, O'Connor T, Roth C, et al. The influence of maternal prenatal and early childhood nutrition and maternal prenatal stress on offspring immune system development and neurodevelopmental disorders. *Frontiers in Neuroscience* 2013;7 doi: 10.3389/fnins.2013.00120
24. Ibanez G, Bernard J, Rondet C, et al. Effects of Antenatal Maternal Depression and Anxiety on Children's Early Cognitive Development: A Prospective Cohort Study. *Plos One* 2015;10(8) doi: 10.1371/journal.pone.0135849
25. Tarabulsy G, Pearson J, Vaillancourt-Morel M, et al. Meta-Analytic Findings of the Relation Between Maternal Prenatal Stress and Anxiety and Child Cognitive Outcome. *Journal of Developmental and Behavioral Pediatrics* 2014;35(1):38-43.
26. Knudsen A, Skogen J, Ystrom E, et al. Maternal pre-pregnancy risk drinking and toddler behavior problems: the Norwegian Mother and Child Cohort Study. *European Child & Adolescent Psychiatry* 2014;23(10):901-11. doi: 10.1007/s00787-014-0588-x
27. Parazzini F, Davoli E, Rabaiotti M, et al. Validity of self-reported smoking habits in pregnancy: a saliva cotinine analysis. *Acta Obstet Gynecol Scand* 1996;75(4):352-4.
28. Dubois J, Dehaene-Lambertz G, Kulikova S, et al. THE EARLY DEVELOPMENT OF BRAIN WHITE MATTER: A REVIEW OF IMAGING STUDIES IN FETUSES, NEWBORNS AND INFANTS. *Neuroscience* 2014;276:48-71. doi: 10.1016/j.neuroscience.2013.12.044
29. Chastang J, Baiz N, Cadwalladder J, et al. Postnatal Environmental Tobacco Smoke Exposure Related to Behavioral Problems in Children. *Plos One* 2015;10(8) doi: 10.1371/journal.pone.0133604
30. Carter S, Paterson J, Gao W, et al. Maternal smoking during pregnancy and behaviour problems in a birth cohort of 2-year-old Pacific children in New Zealand. *Early Hum Dev* 2008;84(1):59-66. doi: 10.1016/j.earlhumdev.2007.03.009 [published Online First: 2007/05/17]
31. Naeye RL, Peters EC. Mental development of children whose mothers smoked during pregnancy. *Obstet Gynecol* 1984;64(5):601-7.
32. Langley K, Holmans PA, van den Bree MB, et al. Effects of low birth weight, maternal smoking in pregnancy and social class on the phenotypic manifestation of Attention Deficit Hyperactivity Disorder and associated antisocial behaviour: investigation in a clinical sample. *BMC Psychiatry* 2007;7:26. doi: 10.1186/1471-244X-7-26 [published Online First: 2007/06/20]
33. Wakschlag LS, Lahey BB, Loeber R, et al. Maternal smoking during pregnancy and the risk of conduct disorder in boys. *Arch Gen Psychiatry* 1997;54(7):670-6.
34. Key AP, Ferguson M, Molfese DL, et al. Smoking during pregnancy affects speech-processing ability in newborn infants. *Environ Health Perspect* 2007;115(4):623-9. doi: 10.1289/ehp.9521 [published Online First: 2006/11/28]

For peer review only - http://bmjopen.bmj.com/site/about/guidelines.xhtml

35. Olds DL, Henderson CR, Tatelbaum R. Intellectual impairment in children of women who smoke cigarettes during pregnancy. *Pediatrics* 1994;93(2):221-7.
36. Mortensen EL, Michaelsen KF, Sanders SA, et al. A dose-response relationship between maternal smoking during late pregnancy and adult intelligence in male offspring. *Paediatr Perinat Epidemiol* 2005;19(1):4-11. doi: 10.1111/j.1365-3016.2004.00622.x
37. Batty GD, Der G, Deary IJ. Effect of maternal smoking during pregnancy on offspring's cognitive ability: empirical evidence for complete confounding in the US national longitudinal survey of youth. *Pediatrics* 2006;118(3):943-50. doi: 10.1542/peds.2006-0168
38. Breslau N, Paneth N, Lucia VC, et al. Maternal smoking during pregnancy and offspring IQ. *Int J Epidemiol* 2005;34(5):1047-53. doi: 10.1093/ije/dyi163 [published Online First: 2005/08/05]
39. Roza SJ, Verhulst FC, Jaddoe VW, et al. Maternal smoking during pregnancy and child behaviour problems: the Generation R Study. *Int J Epidemiol* 2009;38(3):680-9. doi: 10.1093/ije/dyn163 [published Online First: 2008/09/04]
40. Kendall S, Nash A, Braun A, et al. Evaluating the use of a population measure of child development in the Healthy Child Programme Two Year Review, 2014.
41. Yu L, Hey E, Doyle L, et al. Evaluation of the Ages and Stages Questionnaires in identifying children with neurosensory disability in the Magpie Trial follow-up study. *Acta Paediatrica* 2007;96(12):1803-08. doi: 10.1111/j.1651-2227.2007.00517.x

For peer review only

For peer review only - http://bmjopen.bmj.com/site/about/guidelines.xhtml

STROBE 2007 (v4) checklist of items to be included in reports of observational studies in epidemiology*
Checklist for cohort, case-control, and cross-sectional studies (combined)

Section/Topic	Item #	Recommendation	Reported on page #
**Title and abstract**	1	(*a*) Indicate the study's design with a commonly used term in the title or the abstract	1
		(*b*) Provide in the abstract an informative and balanced summary of what was done and what was found	2
**Introduction**			
Background/rationale	2	Explain the scientific background and rationale for the investigation being reported	4
Objectives	3	State specific objectives, including any pre-specified hypotheses	5
**Methods**			
Study design	4	Present key elements of study design early in the paper	5
Setting	5	Describe the setting, locations, and relevant dates, including periods of recruitment, exposure, follow-up, and data collection	5
Participants	6	(*a*) *Cohort study*—Give the eligibility criteria, and the sources and methods of selection of participants. Describe methods of follow-up<br>*Case-control study*—Give the eligibility criteria, and the sources and methods of case ascertainment and control selection. Give the rationale for the choice of cases and controls<br>*Cross-sectional study*—Give the eligibility criteria, and the sources and methods of selection of participants	5
		(*b*) *Cohort study*—For matched studies, give matching criteria and number of exposed and unexposed<br>*Case-control study*—For matched studies, give matching criteria and the number of controls per case	
Variables	7	Clearly define all outcomes, exposures, predictors, potential confounders, and effect modifiers. Give diagnostic criteria, if applicable	5,6,7
Data sources/ measurement	8*	For each variable of interest, give sources of data and details of methods of assessment (measurement). Describe comparability of assessment methods if there is more than one group	*5,6,7*
Bias	9	Describe any efforts to address potential sources of bias	5,6
Study size	10	Explain how the study size was arrived at	
Quantitative variables	11	Explain how quantitative variables were handled in the analyses. If applicable, describe which groupings were chosen and why	5,6,7
Statistical methods	12	(*a*) Describe all statistical methods, including those used to control for confounding	8
		(*b*) Describe any methods used to examine subgroups and interactions	n/a
		(*c*) Explain how missing data were addressed	9
		(*d*) *Cohort study*—If applicable, explain how loss to follow-up was addressed<br>*Case-control study*—If applicable, explain how matching of cases and controls was addressed	

For peer review only

		Cross-sectional study—If applicable, describe analytical methods taking account of sampling strategy	
		(*e*) Describe any sensitivity analyses	
**Results**			
Participants	13*	(a) Report numbers of individuals at each stage of study—eg numbers potentially eligible, examined for eligibility, confirmed eligible, included in the study, completing follow-up, and analysed	9
		(b) Give reasons for non-participation at each stage	9
		(c) Consider use of a flow diagram	9
Descriptive data	14*	(a) Give characteristics of study participants (eg demographic, clinical, social) and information on exposures and potential confounders	9,10,11
		(b) Indicate number of participants with missing data for each variable of interest	11
		(c) *Cohort study*—Summarise follow-up time (eg, average and total amount)	
Outcome data	15*	*Cohort study*—Report numbers of outcome events or summary measures over time	9,10,11,12
		Case-control study—Report numbers in each exposure category, or summary measures of exposure	
		Cross-sectional study—Report numbers of outcome events or summary measures	
Main results	16	(*a*) Give unadjusted estimates and, if applicable, confounder-adjusted estimates and their precision (eg, 95% confidence interval). Make clear which confounders were adjusted for and why they were included	10,11
		(*b*) Report category boundaries when continuous variables were categorized	
		(*c*) If relevant, consider translating estimates of relative risk into absolute risk for a meaningful time period	
Other analyses	17	Report other analyses done—eg analyses of subgroups and interactions, and sensitivity analyses	
**Discussion**			
Key results	18	Summarise key results with reference to study objectives	13
Limitations	19	Discuss limitations of the study, taking into account sources of potential bias or imprecision. Discuss both direction and magnitude of any potential bias	13,14
Interpretation	20	Give a cautious overall interpretation of results considering objectives, limitations, multiplicity of analyses, results from similar studies, and other relevant evidence	13,14,15
Generalisability	21	Discuss the generalisability (external validity) of the study results	15,16
**Other information**			
Funding	22	Give the source of funding and the role of the funders for the present study and, if applicable, for the original study on which the present article is based	17

*Give information separately for cases and controls in case-control studies and, if applicable, for exposed and unexposed groups in cohort and cross-sectional studies.

Note: An Explanation and Elaboration article discusses each checklist item and gives methodological background and published examples of transparent reporting. The STROBE checklist is best used in conjunction with this article (freely available on the Web sites of PLoS Medicine at http://www.plosmedicine.org/, Annals of Internal Medicine at http://www.annals.org/, and Epidemiology at http://www.epidem.com/). Information on the STROBE Initiative is available at www.strobe-statement.org.

For peer review only

BMJ Open

Is the apparently protective effect of maternal nicotine replacement therapy (NRT) used in pregnancy on infant development explained by smoking cessation?: secondary analyses of a randomised controlled trial

Journal:	*BMJ Open*
Manuscript ID	bmjopen-2018-024923.R1
Article Type:	Research
Date Submitted by the Author:	09-Jan-2019
Complete List of Authors:	Iyen, Barbara; University of Nottingham, Division of Primary Care<br>Vaz, Luis; University of Nottingham Division of Primary Care,<br>Taggar, Jaspal; University of Nottingham Faculty of Medicine and Health Sciences, Division of Primary Care, School of Medicine<br>Cooper, Sue; University of Nottingham, Division of Primary Care<br>Lewis, Sarah; University of Nottingham, Division of Epidemiology and Public Health<br>Coleman, Tim; University of Nottingham, Division of Primary Care
**Primary Subject Heading**:	Smoking and tobacco
Secondary Subject Heading:	Epidemiology, Obstetrics and gynaecology, Paediatrics, Public health, Smoking and tobacco
Keywords:	Maternal medicine < OBSTETRICS, Developmental neurology & neurodisability < PAEDIATRICS, EPIDEMIOLOGY

SCHOLARONE™
Manuscripts

Is the apparently protective effect of maternal nicotine replacement therapy (NRT) used in pregnancy on infant development explained by smoking cessation?: secondary analyses of a randomised controlled trial

Barbara Iyen[1], PhD., Luis R. Vaz[1], PhD., Jaspal Taggar[1], PhD., Sue Cooper[1], PhD., Sarah Lewis[2], PhD., Tim Coleman[1],MD.

Affiliations: [1]Division of Primary care, University of Nottingham, UK and [2]Division of Epidemiology and Public health, University of Nottingham, UK

Address correspondence to:
Barbara Iyen
Division of Primary Care
University of Nottingham,
Tower Building, Room 1402, Nottingham
Email: Barbara.iyen@nottingham.ac.uk;
Tel.: +44 115 84 66588
Fax: +44 115 84 66904

Short title:
Maternal exposure to tobacco smoke in pregnancy and infant development at age 2

Keywords:
Smoking in pregnancy, maternal smoking, child development, developmental impairment

Word count (abstract): 300
Word count (text): 3,787

For peer review only

For peer review only - http://bmjopen.bmj.com/site/about/guidelines.xhtml

ABSTRACT

Objective: To investigate relationships between maternal smoking status in pregnancy and infant development. The largest RCT of nicotine replacement therapy (NRT) for smoking cessation in pregnancy, the Smoking and Nicotine in Pregnancy (SNAP) trial, found that at one month after randomisation, smoking cessation rates were doubled in the NRT group than in the placebo group. At delivery, there was no significant difference in cessation rates in both groups. Surprisingly, infants born to women randomised to NRT were more likely to have unimpaired development at 2 years. We hypothesized that this apparently protective effect was due to smoking cessation caused by NRT and so, investigate this relationship using the same cohort

Design: Secondary analysis of a randomised controlled trial

Setting: Seven antenatal hospitals in the Midlands and North-West England

Participants: Eight hundred and eighty four (884) pregnant smokers randomised to receive either NRT patches or visually-identical placebo in the SNAP trial. Participants' smoking behaviour were recorded at randomisation, one month after their target quit date and at delivery.

Methods: Using logistic regression models, we investigated associations between participants' smoking measures and infant development (assessed using the Ages and Stages questionnaire) at 2 years.

Main outcome measures: 2-year infant development

Results: Developmental impairment was reported for 12.7% of study 2 year olds. Maternal heaviness of smoking at randomisation (odds ratio [OR]: 1.26, 95% confidence interval [95% CI]: 0.82-1.96, p = 0.091), validated smoking abstinence recorded at one month after a quit date (OR: 1.02, 95% CI: 0.60-1.74, p = 0.914) and validated smoking abstinence recorded at one month after a quit date and at the end of pregnancy (OR: 1.52, 95% CI: 0.81-2.85, p = 0.795) were not independently associated with infant developmental impairment at 2 years.

For peer review only

For peer review only - http://bmjopen.bmj.com/site/about/guidelines.xhtml

Conclusion: We found no evidence that NRT treatment improved infants' developmental outcomes through smoking cessation.

ARTICLE SUMMARY

Strengths and limitations of this study

- This study uses data from the smoking and nicotine in pregnancy (SNAP) trial, the largest randomised controlled trial of nicotine replacement therapy (NRT) for smoking cessation in Pregnancy.
- The SNAP trial remains the only maternal smoking cessation trial to investigate infant outcomes beyond delivery.
- Baseline characteristics and potential confounders were included in multivariable analyses to determine independent associations between maternal smoking and 2-year developmental outcomes.
- Maternal factors which were not measured in the SNAP trial could not be adjusted for in multivariable analyses.

For peer review only

For peer review only - http://bmjopen.bmj.com/site/about/guidelines.xhtml

INTRODUCTION

Smoking in pregnancy is associated with increased risks of many adverse outcomes including miscarriage, stillbirth, prematurity, low birth weight, perinatal morbidity and mortality[1] and is a substantial international public health problem; in high income countries 13% to 25% of pregnant women smoke [2-5] and in developing countries[6 7], the World Health Organisation predicts a future epidemic. Fortunately, impacts on the fetus are avoidable and cessation in pregnancy improves infants' birth weights and reduces the risk of premature birth.[8] Additionally, pregnancy is the life event which seems to most motivate smokers' cessation attempts; over 50% of UK pregnant smokers try stopping.[5] Women are, therefore, likely to be receptive to support with stopping and, as it is effective for non-pregnant smokers[9] and possibly also for pregnant smokers too, nicotine replacement therapy (NRT) is frequently offered to pregnant women who smoke[10]. Although NRT is not thought to be completely risk-free because it exposes users to nicotine, this is considered safer than smoking because smokers are already nicotine-exposed and NRT users are not exposed to all the numerous tobacco smoke carcinogens.

Animal experiments and human-subject laboratory investigations suggest that nicotine may adversely affect the fetal cardiovascular system and developing nerve tissue[11 12], however trials of NRT for smoking cessation in pregnancy have been permitted because any fetal harm from the nicotine would likely be compensated for by the benefits of mothers stopping smoking[13]. In the largest of these trials, compliance rates in both the NRT and the placebo arms were low, with only 7.2% of women assigned to receive NRT and 2.8% of women assigned to receive placebo, using patches for more than one month. Nicotine patches doubled cessation rates at four weeks but by delivery there was no significant difference in cessation rate between the NRT and placebo groups.[14] Unexpectedly, infants born to NRT group women were 4% more likely to have unimpaired development at two years of age than infants born to women in the placebo groups[15] (OR 1.40, 95% CI: 1.05-1.86, p=0.023) and there was a dose-response relationship between adherence to NRT and

For peer review only - http://bmjopen.bmj.com/site/about/guidelines.xhtml

impairment-free infant development.[15] It seems implausible that this effect would be due to nicotine having a direct beneficial effect on the fetus, and there is a possibility that the observed effect might be due to differences in unmeasured characteristics, between the participants in the NRT group and those in the placebo group. However, as NRT had a substantial impact on cessation in early pregnancy, we hypothesise that the better developmental outcomes in NRT group infants and the dose-response relationship between increasing NRT use and these might be explained by smoking cessation during pregnancy caused by NRT use. Consequently, we present secondary analyses of data from the same trial to investigate whether or not the absence of infants' developmental impairments at two years was associated with maternal smoking status measured at different points in the trial.

METHODS

Study participants

We used data from the Smoking, Nicotine and Pregnancy (SNAP) trial, a placebo-randomised -controlled trial of NRT for smoking cessation in pregnant women[14]. The trial recruited 1,050 participants aged 16-45 years between 12 and 24 weeks gestation who smoked at least ten cigarettes a day before pregnancy and at least five cigarettes a day during pregnancy, with exhaled carbon monoxide (CO) readings of ≥8ppm. Participants were randomised to receive up to an 8-week course of either 15mg per 16 hours of NRT patches or visually-identical placebo. For safety reasons, women were instructed that if they smoked at all whilst using patches, the NRT should be stopped. Participants set a target quit date and were followed up one month after this, during hospital admission for delivery or as soon as possible afterwards, and at 24 months after delivery.

For peer review only - http://bmjopen.bmj.com/site/about/guidelines.xhtml

Maternal baseline data and smoking behaviour measures

Baseline data included date of birth, age on leaving full time education, number of cigarettes smoked per day prior to and during pregnancy, body mass index (BMI), the heaviness of smoking index (HSI) - a measure of nicotine dependence which is a six-point scale derived from the time to first cigarette after waking and the number of cigarettes smoked daily[16]. Other data included were previous use of NRT in the current pregnancy and partners' smoking status.

Self-reported smoking data were obtained one month after the target quit date and at delivery. Self-reported smoking abstinence at one-month after the quit date was validated by exhaled carbon monoxide (CO) measurements below 8ppm while abstinence at delivery was validated by CO measurements and estimated saliva cotinine (COT) concentrations below 10ng/ml. No data on tobacco smoke exposure were collected between these two time-points. Self-reported data on the use of nicotine patches were collected one month after the target quit date and at delivery; adherence to NRT have been reported elsewhere[14].

Infant outcome measures

Birth status (live or still birth), birth weight, singleton or multiple birth, sex and gestational age at birth were recorded at delivery and have been reported elsewhere[14]. The presence of infant developmental impairment at 24 months was determined using questionnaire responses from participants or from healthcare professionals when there was no participant response; full details have been described previously[15]. Participants reported on their infants' development as assessed within the 5 domains of the Ages and Stages Questionnaire edition 3 (ASQ-3): communication, gross motor, fine motor, problem solving and personal-social skills.[15] Participant questionnaires (PQ2) were posted out at 24 months and if no response was received, health professional ones were dispatched to non-respondents' family physicians. Health professional questionnaire (HPQ) items were consistent with domains in participant questionnaires and were designed to be completed with

For peer review only - http://bmjopen.bmj.com/site/about/guidelines.xhtml

reference to medical or health visitor records. Scores from the five ASQ-3 domains and responses to 'non-domain' ASQ-3 items in the participant questionnaires were used with established norms to categorise infants as having 'developmental impairment' or 'no developmental impairment'. Infants with only HPQ responses were considered to have no developmental impairment when the responses from all questions indicated no potential developmental issues.[15] As shown in the main study, 88% of study participants returned a PQ2 (participant questionnaire) or HPQ (health professional questionnaire). The proportion of participants assessed using the PQ2 or HPQ were similar in the treatment arm and placebo arm. Approximately two-thirds of participants completed and returned the PQ2, while the HPQ was the source of data for children of the remaining participants.

Measures of tobacco smoke exposure

As outlined above, smoking behaviour measures were collected at randomisation, one month after a target quit date and at the end of pregnancy. From these data, we derived the following categories to represent different levels of tobacco smoke exposure during pregnancy:

i) Number of cigarettes smoked daily during pregnancy, at randomisation

ii) Heaviness of smoking index (HSI) at randomisation, categorised as high (4-6) or low (0-3)

iii) Women who reported not smoking between their target quit date and one month, validated using exhaled CO.

iv) Women who reported not smoking between their target quit date and the end of pregnancy or shortly afterwards, validated by either or both of exhaled CO and saliva cotinine at the end of pregnancy and also by exhaled CO at one month after their quit date.

As all trial participants smoked at time of randomisation, exposure category (ii) was intended to dichotomise those who smoked more heavily and less heavily in the first weeks of pregnancy prior to study randomisation. For categories (iii) and (iv), we hypothesised that those demonstrating

For peer review only - http://bmjopen.bmj.com/site/about/guidelines.xhtml

validated abstinence at both one month and the end of pregnancy (category iv) would have a lower overall tobacco smoke exposure than those for whom abstinence was only validated at one month after their quit date. However, it should be noted that no biochemical test can validate smoking cessation for a prolonged period and some women in either group may have smoked for a time between baseline / study enrolment and one month or the end of pregnancy.

Analyses

Analyses were carried out for participants who had live singleton births. Univariable logistic regression was used to investigate the association between 2-year developmental outcomes and different measures of maternal tobacco smoke exposure such as baseline number of cigarettes smoked daily in pregnancy, the heaviness of smoking index during pregnancy, maternal smoking abstinence at some point during pregnancy and maternal smoking abstinence during the latter stages of pregnancy. Multivariable logistic regression analyses were then conducted to determine the independent associations between the measures of maternal smoking exposure and infant development outcomes. We used a forward stepwise regression to identify potential confounder variables; we included baseline characteristic variables that were found to be significantly associated with maternal smoking exposures and developmental impairment and we also controlled for variables that are well known to be associated with childhood development. Potential confounders included in the multivariable analyses were: birth weight, maternal age, age at which full time education was completed, maternal BMI and partner's smoking status.

All analyses were performed using Stata 14. Significant associations were defined at the $p \leq 0.05$ significance level.

Patient and Public Involvement

There were no patient or public involvement in the original SNAP study

For peer review only - http://bmjopen.bmj.com/site/about/guidelines.xhtml

RESULTS

From 1,050 pregnant smokers enrolled in the SNAP trial, there were 12 women with twin pregnancies, 14 participants who had fetal deaths, 4 lost to follow-up and 10 who withdrew consent. Of 1,010 women with singleton live births, data on developmental impairment was available for 884 infants. Analyses therefore included maternal and 2-year records of these 884 infants (Figure 1). The self-reported median (IQR) number of daily cigarettes smoked by women prior to pregnancy was 19 (10-60). At the time of recruitment into the study, the reported median (IQR) number of cigarettes smoked daily in pregnancy was 15 (10-20). Of the infants born to participants in the study, 52% were males and less than 10% had low birth weight. Developmental impairment was present in 12.7% of these children. (n=112).

Study participants and singleton infants with 2-year outcome data had similar baseline characteristics to participants reported in the full trial cohort[15]. Aside from the fact that participants who were lost to follow-up had higher mean IMD (Index of multiple deprivation) scores implying that they lived in more deprived areas than participants who had data up to 2 years, there were only minor differences in the baseline characteristics between the participants with 2-year data and those who were lost to follow-up[15].

Figure 1.

For peer review only - http://bmjopen.bmj.com/site/about/guidelines.xhtml

Maternal and infant characteristics

The characteristics of the women and children included in the analyses and their associations with infant developmental impairment at two years are presented in table 1. The majority of participants included in the study (72.4%) were less than 30 years old, 39.9% had a BMI between 18.5-24.9kg/m^2. The average age of leaving full time education was 16.3 years. We used the 'heaviness of smoking index' as a measure of the level of maternal nicotine dependence and 65% of women in the study had low levels of nicotine dependence prior to being recruited in the study. More than two-thirds of the study participants had partners who smoked (67.9%). Eighteen percent (18%) of women were validated as abstinent from smoking at some point during pregnancy (measured one month after their target quit date) and 8.9% remained abstinent at the latter stages of pregnancy until delivery. There was no statistically significant relationship between smoking exposure or abstinence and infant developmental impairment. The only variable that was found to be significantly associated with developmental impairment in univariable analysis was infant sex which was retained in addition to the a priori defined variables, as a confounder in multivariable analyses.

For peer review only

Table 1. Maternal and infant characteristics with univariable association between these and infant developmental impairment (N=884)

	n (%)	Unadjusted OR (95% CI) for developmental impairment	p-value
**Maternal characteristics**			
Maternal age (years) at pregnancy (n=884)			
<20	136 (15.4%)	1.00	0.501
20-24	292 (33.0%)	0.62 (0.34-1.11)	
25-29	212 (24.0%)	0.63 (0.34-1.18)	
30-34	148 (16.7%)	0.95 (0.50-1.80)	
35-39	74 (8.4%)	0.81 (0.36-1.82)	
>40	22 (2.5%)	0.82 (0.22-3.00)	
Maternal BMI* [kg/m^2] (n=847)			
<18.5	23 (2.7%)	0.57 (0.15-2.89)	0.853
18.5-24.9	338 (39.9%)	1.00	
25.0-29.9	252 (29.8%)	0.96 (0.59-1.58)	
>30.0	234(27.6%)	1.13 (0.69-1.83)	
Maternal age of leaving full time education** (n=872)	16.3 (1.5)	1.09 (0.98-1.21)	0.698
Partner's smoking status (n=884)			
Non smoker	214 (24.2%)	1.00	0.899
Smoker	600 (67.9%)	1.05 (0.65-1.69)	
No data on partner's smoking status	70 (7.9%)	1.21 (0.55-2.64)	
Heaviness of smoking index (HSI) at study randomisation (N=884)			
Low index (0-3)	576 (65.2%)	1.00	0.209
High index (4-6)	308 (34.8%)	1.30 (0.87-1.95)	
Daily number of cigarettes smoked at study randomisation^ (n=884)	15.0 (10-20)	1.01 (0.98-1.04)	0.674
Daily number of cigarettes smoked at randomisation in categories (n=884)			
5-9/day	172 (19.5%)	1.00	0.29
10-19/day	482 (54.5%)	1.08 (0.62-1.87)	
>20/day	230 (26.0%)	1.49 (0.82-2.71)	
Maternal abstinence from smoking at some point during pregnancy (n=884)			
Did not abstain	725 (82.0%)	1.00	0.970
Abstained	159 (18.0%)	0.99 (0.59-1.66)	
Maternal abstinence from smoking during latter stages of pregnancy (n=884)			
Did not abstain	805 (91.1%)	1.00	0.176
Abstained	79 (8.9%)	1.55 (0.84-2.87)	
**Infant characteristics**			
Sex of baby (n=884)			
Female	422 (47.7%)	1.00	0.003
Male	462 (52.3%)	1.84 (1.22-2.79)	
Birth weight (n=884)			
<2.5kg	85 (9.6%)	1.00	0.408
2.5-3.0kg	215 (24.3%)	0.56 (0.28-1.13)	
3.0-3.5kg	335 (37.9%)	0.63 (0.33-1.21)	
>3.5kg	249 (28.2%)	0.74 (0.38-1.43)	

*Maternal BMI categories defined as underweight "<18.5", normal weight "18.5-24.9", overweight "25.0-29.9", obese ">30.0"

All values are represented as n (%) unless indicated by ** which denotes mean (SD) or ^ which denotes median (interquartile range)

For peer review only

For peer review only - http://bmjopen.bmj.com/site/about/guidelines.xhtml

Table 2. Multivariate models of the association between maternal tobacco smoke exposure in pregnancy and child developmental impairment at 2 years (analyses restricted to 884 mothers with measures of infant 2-year developmental outcome)

	Odds ratio (95% CI) for developmental impairment at age 2	P-value
**Daily number of cigarettes smoked at study randomisation**		
Unadjusted	1.01 (0.98-1.04)	0.674
Adjusted₸	1.01 (0.98-1.05)	0.099
**Maternal heaviness of smoking index at study randomisation (high vs low)**		
Unadjusted	1.30 (0.87-1.95)	0.209
Adjusted₸	1.26 (0.82-1.96)	0.091
**Maternal abstinence from smoking at some point during pregnancy**		
Unadjusted	0.99 (0.59-1.66)	0.970
Adjusted₸	1.02 (0.60-1.74)	0.914
**Maternal abstinence from smoking during latter stages of pregnancy**		
Unadjusted	1.55 (0.84-2.87)	0.176
Adjusted₸	1.52 (0.81-2.85)	0.795

₸ Adjusted for infant birthweight, infant sex, maternal age, age of leaving full time education, maternal BMI and partners' smoking status

The multivariable analyses of the associations between different measures of maternal tobacco smoke exposure and infant developmental impairment are presented in table 2. There remained no statistically significant associations between maternal heaviness of smoking or abstinence from tobacco smoke exposure and infant developmental impairment at two years. The odds ratio for child developmental impairment in infants of mothers who abstained from smoking at some point during pregnancy and in infants of mothers who abstained during the latter stages of pregnancy until delivery, were 0.99 (95% CI 0.59-1.66) and 1.55 (95% CI 0.84-2.87) respectively. On adjusting for measured co-variates and a priori variables, these estimates remained statistically non-significant with odds ratios of 1.02 (95% CI 0.60-1.74) and 1.52 (95% CI 0.81-2.85) respectively.

For peer review only - http://bmjopen.bmj.com/site/about/guidelines.xhtml

DISCUSSION

Summary of principal findings

We found no associations between maternal smoking status at different points during pregnancy and infants' developmental impairment at two years. Consequently, there is no evidence to support the hypothesis that the better infant development observed within infants born to women who were randomised to NRT in the Smoking, Nicotine and Pregnancy (SNAP) trial resulted from smoking cessation induced by nicotine patch use.

Strengths and limitations

Data for this study are from the largest randomised controlled trial of NRT in pregnancy in which participants, health care and research staff were masked to treatment allocation[14]. Follow-up and outcome ascertainment rates were high and there were only minor differences in characteristics of participants lost to follow up and those remaining in the study at 2 years[15]. It remains the only trial of a smoking cessation intervention in pregnancy to monitor infant outcomes; as such it is the only source of data which could be used to investigate the study hypothesis.

The ASQ-3 is a well-validated screening tool which has a sensitivity of 100% and specificity of 93% for detecting severe developmental delay at 24 months[17]. While some study infants may have been falsely identified as developmentally impaired, there is no reason to believe that such ASQ-3 false positives would have occurred more frequently in infants of mothers who smoked in pregnancy. Whist developmental impairment was assessed in some infants using the participant questionnaire, others had the health professional questionnaire as the main source of data on impairment. Although this consequently implies a non-standardised assessment of the outcome in all infants, findings from the main study had shown that the proportion of participants assessed with either the PQ2 or the HPQ were similar in the treatment and placebo arms. Also there were similarities in the

For peer review only - http://bmjopen.bmj.com/site/about/guidelines.xhtml

baseline characteristics and birth outcomes of participants who returned the PQ2 and those for whom the HPQs were completed.

Although we adjusted for some potentially confounding factors which are known to be associated with infant development such as maternal socioeconomic status[18], maternal education[19], low birth weight[20], maternal exposure to passive smoking[21] and maternal obesity[22] ,we were unable to adjust for those on which we had no data, such as maternal nutrition[23], depressive illness[24], stress and anxiety[25] and alcohol consumption[26] which could also potentially influence infant development but which were not measured in the SNAP trial. There remains therefore, the potential for residual confounding. However, no analyses had borderline significant results so such confounding would need to have substantial impact to mask associations and this seems unlikely.

We undertook secondary analyses of randomised controlled trial data and the original sample size was intended to have sufficient power only for detecting differences in validated smoking cessation rates at the end of pregnancy. Analyses presented here may not have sufficient power to demonstrate associations between smoking and infant development; however one would expect to find one or more such associations if, the relationship between NRT use and infant development which was demonstrated within the same data, were explained by smoking cessation caused by NRT.

There may have been some misattribution of smoking status. Measures of smoking status used were biochemically-validated at two different time points in pregnancy. Validation with carbon monoxide at the earlier time point could only eliminate smoking within the previous 24 hours; and at delivery, with saliva could only do so for the previous seven days. Consequently, some participants may have relapsed or smoked occasionally between these times[27]; however we believe it is reasonable to assume that women who were validated as not smoking both at the end of pregnancy and at one month after randomisation would have lower overall tobacco smoke exposure than those who were not. As recruitment into the study occurred between 12 and 24 weeks gestation, the timing of

For peer review only - http://bmjopen.bmj.com/site/about/guidelines.xhtml

smoking status measurements in relation to gestation will have differed between participants but there is no evidence that smoking is more or less harmful at any point in pregnancy. Although these were the best available data for analyses, the assumptions outlined above need to be considered when interpreting study findings.

Our analyses did not incorporate postnatal environmental cigarette smoke exposure which may have an independent influence on infant development[28]. We know that there is continued maturation of the connections between brain regions after birth, with intense brain white matter myelination in the first post-natal months which becomes progressively less rapid through toddlerhood until young adulthood[29]. Additionally, postnatal exposure to environmental tobacco is associated with emotional and conduct behavioural problems in school aged children[30] and so may have an aetiological role in infant developmental impairment. As maternal prenatal and postnatal smoking are strongly correlated, this would increase the likelihood of finding associations between smoking in pregnancy and infant development; as none were found there is no suggestion of a large impact on findings.

Comparison with literature

As this is an original analysis in a unique trial database there is very little literature with which study findings can be compared. Previous observational studies have found prenatal tobacco exposure to be associated with offspring adverse consequences including behavioural[31] and hyperactivity[32 33] problems, language and reading deficits[32], conduct disorders[34], newborn basic perceptual skills[35] and intellectual impairment in childhood[36] and later life[37]. However, other studies which adjusted for potentially confounding factors such as maternal education [38 39] and parental socioeconomic status[40]have not. Consistent with these latter studies, our findings found no evidence that the improved developmental outcomes in infants of pregnant smokers who used NRT in the SNAP trial[15] were explained by smoking cessation. As pre-clinical studies show that nicotine is neurotoxic and can adversely affect the developing central nervous system[11 12], it also seems unlikely that these better

For peer review only - http://bmjopen.bmj.com/site/about/guidelines.xhtml

outcomes are due to a direct protective effect of NRT on the developing fetus. Perhaps, it is possible that nicotine used in pregnancy has no impact on infants' developmental outcomes and the apparently protective effect of NRT on developmental outcomes observed in the SNAP trial occurred by chance.

The strong univariate association between male infants and developmental impairment was an interesting and unexpected finding which has been suggested in previous studies[41 42]. The ASQ-3 is utilised as a gender-neutral tool however certain questions in the tool have been perceived by health professionals and parents to be gender-oriented and so, thought to potentially elicit more positive responses about either boys or girls[43]. In its routine application as part of the two-year health and development review, the ASQ-3 has been shown to be valid in identifying infants at risk of developmental impairment irrespective of gender[17 44]. To assess if our 'gender-developmental impairment' association was due to a gender-specific impact of nicotine from maternal smoking, we performed a sensitivity analysis of this association adjusting for maternal tobacco exposure, but the association was unchanged and remained strong. We are certain that the earlier finding of better developmental outcomes among infants born to women randomised to the NRT arm of the SNAP trial is not explained by gender, as the proportion of male infants born to participants randomised to the NRT and placebo groups were similar at 49% and 51% respectively.

CONCLUSION

We found no evidence that the apparently protective effect of NRT patch use in pregnancy on infant development two years after delivery as seen in the Smoking, Nicotine and Pregnancy (SNAP) trial, was due to the smoking cessation in pregnancy caused by NRT.

For peer review only

ACKNOWLEDGEMENTS

We gratefully acknowledge the SNAP trial team members. In addition to listed authors, the complete trial team includes: Investigators : Kim Watts, Jim Thornton, Jo Leonardi-Bee, John Britton, Paul Aveyard, Michael Coughtrie, Christine Godfrey, Clare Mannion and Neil Marlow. Research staff: Janet Brown, Yvette Davis, Anne Dickinson, Caroline Dixon, Fiona Holloway, Joanne Lakin, Jayne Platts, Farzana Rashid, Amanda Redford, Cara Taylor. Principal investigators (in recruiting centres): Jonathan Allsop, Simon Cunningham, Karen Glass, Vince Hall, Khaled Ismail, Margaret Ramsay. Midwife leads (in recruiting centres): Sheena Appleby. Denise Bailey, Linda Gustard, Emma Haworth, Grace Hopps, Amanda Lindley, Chris Kettle, Colleen Pearce, Dymphna Sexton-Bradshaw, Julia Savage, Sandra Smith, Sheila Taylor, Alison Witham. Primary Care Trust & NHS Stop Smoking Services' Staff : Barbara Brady, Michelle Battlemuch, Wendy Dudley, Rochelle Edwards, Lorraine Frith, Indu Hari, Catriona Holden, Linda Hoskyns, Paul Jackson, Giri Rajaratnam, Deborah Richardson, Lucy Wade, Maureen Whittaker. QMC Pharmacy: Bernie Cook, Sheila Hodgson (Lead pharmacist), Lisa Humphries, Bernie Sanders (Qualified Person). University of Nottingham Clinical Trials Unit: Dan Simpkins. University of Dundee: Sheila Sharp.

COMPETING INTERESTS

The authors have no conflicts of interest relevant to this study.

FUNDING

This work was supported by the National Institute for Health Research (NIHR) Collaboration for Leadership in Applied Health Research and Care (CLAHRC) East Midlands at NHS Nottingham City CCG. BI's clinical academic lectureship is fully funded by the NIHR. TC, LV, SC and, SL, and TC are members of the UK Centre for Tobacco and Alcohol Studies (UKCTAS). TC, LV, SC and JT are members of the NIHR School for Primary care Research. Professor Coleman is a National Institute for Health Research Senior Investigator. The views and opinions expressed are those of the authors and do not necessarily reflect those of the NIHR Health Technology Assessment (HTA) Programme, the National Health Service (NHS), the NIHR or the Department of Health.

For peer review only - http://bmjopen.bmj.com/site/about/guidelines.xhtml

DETAILS OF ETHICS APPROVAL

National research ethical approval for the SNAP trial was granted by Oxfordshire Research Ethics Committee A, and additional local approvals for each recruitment centre and Clinical Trial Authorisation (CTA) approval was from the MHRA (CTA number: 03057/0002/001-0001). All participants gave written informed consent, including access to medical records for maintaining contact and for the follow-up of their child's health status.

AUTHOR STATEMENT

BI had full access to all of the data in the study and takes responsibility for the integrity of the data and the accuracy of the analyses. BI also wrote the first draft of the manuscript. LV contributed to the initial stages of data organisation, study concept and design, and proofread and approved the full manuscript prior to submission. TC contributed to the study hypothesis and design, data interpretation and analyses, assisted with writing up the "introduction" section and proofread and approved the final manuscript prior to submission. JT, SL and SC contributed to the data interpretation and analyses of this study as well as revision of the manuscript prior to submission.

DATA SHARING STATEMENT

The study protocol for the SNAP trial is publicly available online. Requests for access to de-identified individual participant data that underlie the results reported in this article should be made to the authors of the original study.

For peer review only

For peer review only - http://bmjopen.bmj.com/site/about/guidelines.xhtml

Figure legends

Figure 1. Flow diagram of study participants

Participants with twin pregnancies (n=12), fetal deaths (n=14), loss to follow-up (n=4) and those who withdrew consent (n=10) were excluded from the study analyses. Among participants with singleton live births, there were no data on 2-year infant outcomes for 126 participants, so these participants were also excluded from the analyses.

For peer review only

For peer review only - http://bmjopen.bmj.com/site/about/guidelines.xhtml

REFERENCES

1. Smoking and the young. A report of a working party of the Royal College of Physicians. London: RCP, 1992.

2. Mohsin M, Bauman AE. Socio-demographic factors associated with smoking and smoking cessation among 426,344 pregnant women in New South Wales, Australia. *Bmc Public Health* 2005;5 doi: Artn 138 Doi 10.1186/1471-2458-5-138

3. Jensen KE, Jensen A, Nohr B, et al. Do pregnant women still smoke? A study of smoking patterns among 261,029 primiparous women in Denmark 1997-2005. *Acta Obstetricia Et Gynecologica Scandinavica* 2008;87(7):760-67. doi: Doi 10.1080/00016340802179814

4. Centers for Disease Control and Prevention. Smoking during pregnancy--United States, 1990-2002. *MMWR Morb Mortal Wkly Rep* 2004;39(53):4.

5. McAndrew F, Thompson J, Fellows L, et al. Infant Feeding Survey 2010: Health and Social Care Information Centre, 2012.

6. Oncken CA, Dietz PM, Tong VT, et al. Prenatal tobacco prevention and cessation interventions for women in low- and middle-income countries. *Acta Obstet Gynecol Scand* 2010;89(4):442-53. doi: 10.3109/00016341003678450 [published Online First: 2010/03/20]

7. World Health Organization. Report on the Global Tobacco Epidemic 2008—the mpower package. Geneva: World Health Organization, 2008.

8. Chamberlain C, O'Mara-Eves A, Oliver S, et al. Psychosocial interventions for supporting women to stop smoking in pregnancy. *Cochrane Database Syst Rev* 2013;10:CD001055. doi: 10.1002/14651858.CD001055.pub4

9. Stead LF, Perera R, Bullen C, et al. Nicotine replacement therapy for smoking cessation. *Cochrane Database Syst Rev* 2012;11:CD000146. doi: 10.1002/14651858.CD000146.pub4

For peer review only - http://bmjopen.bmj.com/site/about/guidelines.xhtml

10. Dhalwani NN, Szatkowski L, Coleman T, et al. Prescribing of nicotine replacement therapy in and around pregnancy: a population-based study using primary care data. *Br J Gen Pract* 2014;64(626):e554-60. doi: 10.3399/bjgp14X681361

11. Dempsey DA, Benowitz NL. Risks and benefits of nicotine to aid smoking cessation in pregnancy. [Review] [340 refs]. *Drug Safety* 2001;24(4):277-322.

12. Benowitz N, Dempsey D. Pharmacotherapy for smoking cessation during pregnancy. *Nicotine & Tobacco Research* 2004;6 Suppl 2:S189-202. doi: 10.1080/14622200410001669169 [published Online First: 2004/06/19]

13. Benowitz NL, Dempsey DA, Goldenberg RL, et al. The use of pharmacotherapies for smoking cessation during pregnancy. *Tobacco Control* 2000;9 Suppl 3:III91-III94.

14. Coleman T, Cooper S, Thornton JG, et al. A randomized trial of nicotine-replacement therapy patches in pregnancy. *New England Journal of Medicine* 2012;366(9):808-18. doi: http://dx.doi.org/10.1056/NEJMoa1109582

15. Cooper S, Taggar J, Lewis S, et al. Effect of nicotine patches in pregnancy on infant and maternal outcomes at 2 years: follow-up from the randomised, double-blind, placebo-controlled SNAP trial. *The Lancet Respiratory Medicine* 2014;2(9):728-37. doi: http://dx.doi.org/10.1016/S2213-2600(14)70157-2

16. Heatherton TF, Kozlowski LT, Frecker RC, et al. Measuring the heaviness of smoking: using self-reported time to the first cigarette of the day and number of cigarettes smoked per day. *British Journal of Addiction* 1989;84(7):791-99.

17. Gollenberg A, Lynch C, Jackson L, et al. Concurrent validity of the parent-completed Ages and Stages Questionnaires, 2nd Ed. with the Bayley Scales of Infant Development II in a low-risk sample. *Child Care Health and Development* 2010;36(4):485-90. doi: 10.1111/j.1365-2214.2009.01041.x

For peer review only - http://bmjopen.bmj.com/site/about/guidelines.xhtml

18. Ronfani L, Brumatti L, Mariuz M, et al. The Complex Interaction between Home Environment, Socioeconomic Status, Maternal IQ and Early Child Neurocognitive Development: A Multivariate Analysis of Data Collected in a Newborn Cohort Study. *Plos One* 2015;10(5) doi: 10.1371/journal.pone.0127052

19. Carneiro P, Meghir C, Parey M. MATERNAL EDUCATION, HOME ENVIRONMENTS, AND THE DEVELOPMENT OF CHILDREN AND ADOLESCENTS. *Journal of the European Economic Association* 2013;11:123-60. doi: 10.1111/j.1542-4774.2012.01096.x

20. Stein R, Siegel M, Bauman L. Are children of moderately low birth weight at increased risk for poor health? A new look at an old question. *Pediatrics* 2006;118(1):217-23. doi: 10.1542/peds.2005-2836

21. Jorge JG, Botelho C, Silva AM, et al. Influence of passive smoking on learning in elementary school. *J Pediatr (Rio J)* 2016;92(3):260-7. doi: 10.1016/j.jped.2015.08.010

22. Wylie A, Sundaram R, Kus C, et al. Maternal Prepregnancy Obesity and Achievement of Infant Motor Developmental Milestones in the Upstate KIDS Study. *Obesity* 2015;23(4):907-13. doi: 10.1002/oby.21040

23. Marques A, O'Connor T, Roth C, et al. The influence of maternal prenatal and early childhood nutrition and maternal prenatal stress on offspring immune system development and neurodevelopmental disorders. *Frontiers in Neuroscience* 2013;7 doi: 10.3389/fnins.2013.00120

24. Ibanez G, Bernard J, Rondet C, et al. Effects of Antenatal Maternal Depression and Anxiety on Children's Early Cognitive Development: A Prospective Cohort Study. *Plos One* 2015;10(8) doi: 10.1371/journal.pone.0135849

For peer review only

For peer review only - http://bmjopen.bmj.com/site/about/guidelines.xhtml

25. Tarabulsy G, Pearson J, Vaillancourt-Morel M, et al. Meta-Analytic Findings of the Relation Between Maternal Prenatal Stress and Anxiety and Child Cognitive Outcome. *Journal of Developmental and Behavioral Pediatrics* 2014;35(1):38-43.

26. Knudsen A, Skogen J, Ystrom E, et al. Maternal pre-pregnancy risk drinking and toddler behavior problems: the Norwegian Mother and Child Cohort Study. *European Child & Adolescent Psychiatry* 2014;23(10):901-11. doi: 10.1007/s00787-014-0588-x

27. Parazzini F, Davoli E, Rabaiotti M, et al. Validity of self-reported smoking habits in pregnancy: a saliva cotinine analysis. *Acta Obstet Gynecol Scand* 1996;75(4):352-4.

28. Yolton K, Dietrich K, Auinger P, et al. Exposure to environmental tobacco smoke and cognitive abilities among U.S. children and adolescents. *Environmental health perspectives* 2005;113(1):98-103. doi: 10.1289/ehp.7210 [published Online First: 10/07]

29. Dubois J, Dehaene-Lambertz G, Kulikova S, et al. THE EARLY DEVELOPMENT OF BRAIN WHITE MATTER: A REVIEW OF IMAGING STUDIES IN FETUSES, NEWBORNS AND INFANTS. *Neuroscience* 2014;276:48-71. doi: 10.1016/j.neuroscience.2013.12.044

30. Chastang J, Baiz N, Cadwalladder J, et al. Postnatal Environmental Tobacco Smoke Exposure Related to Behavioral Problems in Children. *Plos One* 2015;10(8) doi: 10.1371/journal.pone.0133604

31. Carter S, Paterson J, Gao W, et al. Maternal smoking during pregnancy and behaviour problems in a birth cohort of 2-year-old Pacific children in New Zealand. *Early Hum Dev* 2008;84(1):59-66. doi: 10.1016/j.earlhumdev.2007.03.009 [published Online First: 2007/05/17]

32. Naeye RL, Peters EC. Mental development of children whose mothers smoked during pregnancy. *Obstet Gynecol* 1984;64(5):601-7.

For peer review only

For peer review only - http://bmjopen.bmj.com/site/about/guidelines.xhtml

33. Langley K, Holmans PA, van den Bree MB, et al. Effects of low birth weight, maternal smoking in pregnancy and social class on the phenotypic manifestation of Attention Deficit Hyperactivity Disorder and associated antisocial behaviour: investigation in a clinical sample. *BMC Psychiatry* 2007;7:26. doi: 10.1186/1471-244X-7-26 [published Online First: 2007/06/20]

34. Wakschlag LS, Lahey BB, Loeber R, et al. Maternal smoking during pregnancy and the risk of conduct disorder in boys. *Arch Gen Psychiatry* 1997;54(7):670-6.

35. Key AP, Ferguson M, Molfese DL, et al. Smoking during pregnancy affects speech-processing ability in newborn infants. *Environ Health Perspect* 2007;115(4):623-9. doi: 10.1289/ehp.9521 [published Online First: 2006/11/28]

36. Olds DL, Henderson CR, Tatelbaum R. Intellectual impairment in children of women who smoke cigarettes during pregnancy. *Pediatrics* 1994;93(2):221-7.

37. Mortensen EL, Michaelsen KF, Sanders SA, et al. A dose-response relationship between maternal smoking during late pregnancy and adult intelligence in male offspring. *Paediatr Perinat Epidemiol* 2005;19(1):4-11. doi: 10.1111/j.1365-3016.2004.00622.x

38. Batty GD, Der G, Deary IJ. Effect of maternal smoking during pregnancy on offspring's cognitive ability: empirical evidence for complete confounding in the US national longitudinal survey of youth. *Pediatrics* 2006;118(3):943-50. doi: 10.1542/peds.2006-0168

39. Breslau N, Paneth N, Lucia VC, et al. Maternal smoking during pregnancy and offspring IQ. *Int J Epidemiol* 2005;34(5):1047-53. doi: 10.1093/ije/dyi163 [published Online First: 2005/08/05]

40. Roza SJ, Verhulst FC, Jaddoe VW, et al. Maternal smoking during pregnancy and child behaviour problems: the Generation R Study. *Int J Epidemiol* 2009;38(3):680-9. doi: 10.1093/ije/dyn163 [published Online First: 2008/09/04]

For peer review only

For peer review only - http://bmjopen.bmj.com/site/about/guidelines.xhtml

41. Huang J, Zhu T, Qu Y, et al. Prenatal, Perinatal and Neonatal Risk Factors for Intellectual Disability: A Systemic Review and Meta-Analysis. *PLoS ONE* 2016;11(4):e0153655-e55. doi: 10.1371/journal.pone.0153655

42. Zambrana I, Pons F, Eadie P, et al. Trajectories of language delay from age 3 to 5: Persistence, recovery and late onset2013.

43. Kendall S, Nash A, Braun A, et al. Evaluating the use of a population measure of child development in the Healthy Child Programme Two Year Review, 2014.

44. Yu L, Hey E, Doyle L, et al. Evaluation of the Ages and Stages Questionnaires in identifying children with neurosensory disability in the Magpie Trial follow-up study. *Acta Paediatrica* 2007;96(12):1803-08. doi: 10.1111/j.1651-2227.2007.00517.x

For peer review only

For peer review only - http://bmjopen.bmj.com/site/about/guidelines.xhtml

Figure 1. Flow diagram of study participants

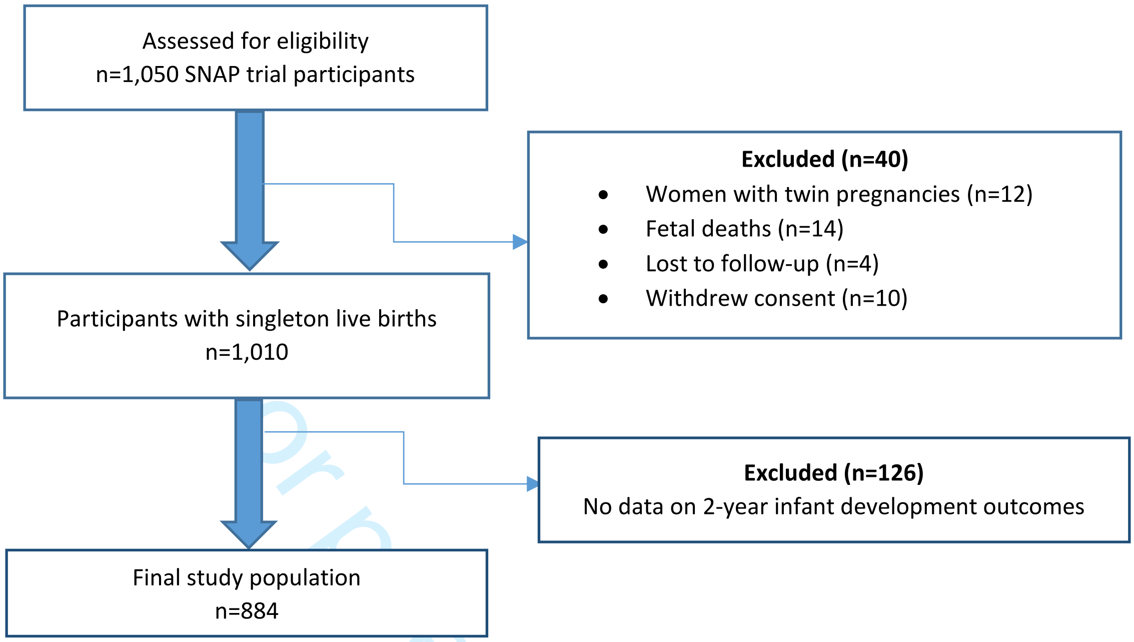

For peer review only

STROBE 2007 (v4) checklist of items to be included in reports of observational studies in epidemiology*
Checklist for cohort, case-control, and cross-sectional studies (combined)

Section/Topic	Item #	Recommendation	Reported on page #
**Title and abstract**	1	(*a*) Indicate the study's design with a commonly used term in the title or the abstract	1
		(*b*) Provide in the abstract an informative and balanced summary of what was done and what was found	2
**Introduction**			
Background/rationale	2	Explain the scientific background and rationale for the investigation being reported	4
Objectives	3	State specific objectives, including any pre-specified hypotheses	5
**Methods**			
Study design	4	Present key elements of study design early in the paper	5
Setting	5	Describe the setting, locations, and relevant dates, including periods of recruitment, exposure, follow-up, and data collection	5
Participants	6	(*a*) *Cohort study*—Give the eligibility criteria, and the sources and methods of selection of participants. Describe methods of follow-up<br>*Case-control study*—Give the eligibility criteria, and the sources and methods of case ascertainment and control selection. Give the rationale for the choice of cases and controls<br>*Cross-sectional study*—Give the eligibility criteria, and the sources and methods of selection of participants	5
		(*b*) *Cohort study*—For matched studies, give matching criteria and number of exposed and unexposed<br>*Case-control study*—For matched studies, give matching criteria and the number of controls per case	
Variables	7	Clearly define all outcomes, exposures, predictors, potential confounders, and effect modifiers. Give diagnostic criteria, if applicable	5,6,7
Data sources/ measurement	8*	For each variable of interest, give sources of data and details of methods of assessment (measurement). Describe comparability of assessment methods if there is more than one group	*5,6,7*
Bias	9	Describe any efforts to address potential sources of bias	5,6
Study size	10	Explain how the study size was arrived at	
Quantitative variables	11	Explain how quantitative variables were handled in the analyses. If applicable, describe which groupings were chosen and why	5,6,7
Statistical methods	12	(*a*) Describe all statistical methods, including those used to control for confounding	8
		(*b*) Describe any methods used to examine subgroups and interactions	n/a
		(*c*) Explain how missing data were addressed	9
		(*d*) *Cohort study*—If applicable, explain how loss to follow-up was addressed<br>*Case-control study*—If applicable, explain how matching of cases and controls was addressed	

For peer review only

		Cross-sectional study—If applicable, describe analytical methods taking account of sampling strategy	
		(*e*) Describe any sensitivity analyses	
**Results**			
Participants	13*	(a) Report numbers of individuals at each stage of study—eg numbers potentially eligible, examined for eligibility, confirmed eligible, included in the study, completing follow-up, and analysed	9
		(b) Give reasons for non-participation at each stage	9
		(c) Consider use of a flow diagram	9
Descriptive data	14*	(a) Give characteristics of study participants (eg demographic, clinical, social) and information on exposures and potential confounders	9,10,11
		(b) Indicate number of participants with missing data for each variable of interest	11
		(c) *Cohort study*—Summarise follow-up time (eg, average and total amount)	
Outcome data	15*	*Cohort study*—Report numbers of outcome events or summary measures over time	9,10,11,12
		Case-control study—Report numbers in each exposure category, or summary measures of exposure	
		Cross-sectional study—Report numbers of outcome events or summary measures	
Main results	16	(*a*) Give unadjusted estimates and, if applicable, confounder-adjusted estimates and their precision (eg, 95% confidence interval). Make clear which confounders were adjusted for and why they were included	10,11
		(*b*) Report category boundaries when continuous variables were categorized	
		(*c*) If relevant, consider translating estimates of relative risk into absolute risk for a meaningful time period	
Other analyses	17	Report other analyses done—eg analyses of subgroups and interactions, and sensitivity analyses	
**Discussion**			
Key results	18	Summarise key results with reference to study objectives	13
Limitations	19	Discuss limitations of the study, taking into account sources of potential bias or imprecision. Discuss both direction and magnitude of any potential bias	13,14
Interpretation	20	Give a cautious overall interpretation of results considering objectives, limitations, multiplicity of analyses, results from similar studies, and other relevant evidence	13,14,15
Generalisability	21	Discuss the generalisability (external validity) of the study results	15,16
**Other information**			
Funding	22	Give the source of funding and the role of the funders for the present study and, if applicable, for the original study on which the present article is based	17

*Give information separately for cases and controls in case-control studies and, if applicable, for exposed and unexposed groups in cohort and cross-sectional studies.

Note: An Explanation and Elaboration article discusses each checklist item and gives methodological background and published examples of transparent reporting. The STROBE checklist is best used in conjunction with this article (freely available on the Web sites of PLoS Medicine at http://www.plosmedicine.org/, Annals of Internal Medicine at http://www.annals.org/, and Epidemiology at http://www.epidem.com/). Information on the STROBE Initiative is available at www.strobe-statement.org.

For peer review only

BMJ Open

Is the apparently protective effect of maternal nicotine replacement therapy (NRT) used in pregnancy on infant development explained by smoking cessation?: secondary analyses of a randomised controlled trial

Journal:	*BMJ Open*
Manuscript ID	bmjopen-2018-024923.R2
Article Type:	Research
Date Submitted by the Author:	11-Mar-2019
Complete List of Authors:	Iyen, Barbara; University of Nottingham University Park Campus, Division of Primary Care<br>Vaz, Luis; University of Nottingham Division of Primary Care<br>Taggar, Jaspal; University of Nottingham Faculty of Medicine and Health Sciences, Division of Primary Care, School of Medicine<br>Cooper, Sue; University of Nottingham University Park Campus, Division of Primary care<br>Lewis, Sarah; University of Nottingham, Division of Epidemiology and Public Health<br>Coleman, Tim; University of Nottingham, Division of Primary Care
**Primary Subject Heading**:	Smoking and tobacco
Secondary Subject Heading:	Epidemiology, Obstetrics and gynaecology, Paediatrics, Public health, Smoking and tobacco
Keywords:	Maternal medicine < OBSTETRICS, Developmental neurology & neurodisability < PAEDIATRICS, EPIDEMIOLOGY

SCHOLARONE™
Manuscripts

Is the apparently protective effect of maternal nicotine replacement therapy (NRT) used in pregnancy on infant development explained by smoking cessation?: secondary analyses of a randomised controlled trial

Barbara Iyen[1], PhD., Luis R. Vaz[1], PhD., Jaspal Taggar[1], PhD., Sue Cooper[1], PhD., Sarah Lewis[2], PhD., Tim Coleman[1],MD.

Affiliations: [1]Division of Primary care, University of Nottingham, UK and [2]Division of Epidemiology and Public health, University of Nottingham, UK

Address correspondence to:
Barbara Iyen
Division of Primary Care
University of Nottingham,
Tower Building, Room 1402, Nottingham
Email: Barbara.iyen@nottingham.ac.uk;
Tel.: +44 115 84 66588
Fax: +44 115 84 66904

Short title:
Maternal exposure to tobacco smoke in pregnancy and infant development at age 2

Keywords:
Smoking in pregnancy, maternal smoking, child development, developmental impairment

Word count (abstract): 298
Word count (text): 3,854

For peer review only - http://bmjopen.bmj.com/site/about/guidelines.xhtml

ABSTRACT

Objective: To investigate relationships between maternal smoking status in pregnancy and infant development. The largest RCT of nicotine replacement therapy (NRT) for smoking cessation in pregnancy, the Smoking and Nicotine in Pregnancy (SNAP) trial, found that at one month after randomisation, smoking cessation rates were doubled in the NRT group compared to the placebo group. At delivery, there was no significant difference in cessation rates between groups. Surprisingly, infants born to women randomised to NRT were more likely to have unimpaired development at 2 years. We hypothesized that this apparently protective effect was due to smoking cessation caused by NRT and so, investigate this relationship using the same cohort.

Design: Secondary analysis of a randomised controlled trial

Setting: Seven antenatal hospitals in the Midlands and North-West England

Participants: Eight hundred and eighty-four (884) pregnant smokers randomised to receive either NRT patches or visually-identical placebo in the SNAP trial. Participants' smoking behaviour were recorded at randomisation, one month after their target quit date and at delivery.

Methods: Using logistic regression models, we investigated associations between participants' smoking measures and infant development (assessed using the Ages and Stages questionnaire) at 2 years.

Main outcome measures: 2-year infant development

Results: Developmental impairment was reported for 12.7% of study 2 year olds. Maternal heaviness of smoking at randomisation (odds ratio [OR]: 1.26, 95% confidence interval [95% CI]: 0.82-1.96, p = 0.091), validated smoking abstinence recorded at one month after a quit date (OR: 1.02, 95% CI: 0.60-1.74, p = 0.914) and validated smoking abstinence recorded at one month after a quit date and at the end of pregnancy (OR: 1.52, 95% CI: 0.81-2.85, p = 0.795) were not independently associated with infant developmental impairment at 2 years.

For peer review only - http://bmjopen.bmj.com/site/about/guidelines.xhtml

Conclusion: We found no evidence that NRT treatment improved infants' developmental outcomes through smoking cessation.

ARTICLE SUMMARY

Strengths and limitations of this study

- This study uses data from the smoking and nicotine in pregnancy (SNAP) trial, the largest randomised controlled trial of nicotine replacement therapy (NRT) for smoking cessation in Pregnancy.
- The SNAP trial remains the only maternal smoking cessation trial to investigate infant outcomes beyond delivery.
- Baseline characteristics and potential confounders were included in multivariable analyses to determine independent associations between maternal smoking and 2-year developmental outcomes.
- Maternal factors which were not measured in the SNAP trial could not be adjusted for in multivariable analyses.

For peer review only

For peer review only - http://bmjopen.bmj.com/site/about/guidelines.xhtml

INTRODUCTION

Smoking in pregnancy is associated with increased risks of many adverse outcomes including miscarriage, stillbirth, prematurity, low birth weight, perinatal morbidity and mortality[1] and is a substantial international public health problem; in high income countries 13% to 25% of pregnant women smoke [2-5] and in developing countries[6 7], the World Health Organisation predicts a future epidemic. Fortunately, impacts on the fetus are avoidable and cessation in pregnancy improves infants' birth weights and reduces the risk of premature birth.[8] Additionally, pregnancy is the life event which seems to most motivate smokers' cessation attempts; over 50% of UK pregnant smokers try stopping.[5] Women are, therefore, likely to be receptive to support with stopping and, as it is effective for non-pregnant smokers[9] and possibly also for pregnant smokers too, nicotine replacement therapy (NRT) is frequently offered to pregnant women who smoke[10]. Although NRT is not thought to be completely risk-free because it exposes users to nicotine, this is considered safer than smoking because smokers are already nicotine-exposed and NRT users are not exposed to the numerous harmful chemicals and tobacco smoke carcinogens such as carbon monoxide, tar and lead.

Animal experiments and human-subject laboratory investigations suggest that nicotine may adversely affect the fetal cardiovascular system and developing nerve tissue[11 12], however trials of NRT for smoking cessation in pregnancy have been permitted because any fetal harm from the nicotine would likely be compensated for by the benefits of mothers stopping smoking[13]. In the largest of these trials, compliance rates in both the NRT and the placebo arms were low, with only 7.2% of women assigned to receive NRT and 2.8% of women assigned to receive placebo, using patches for more than one month. Nicotine patches doubled cessation rates at four weeks but by delivery there was no significant difference in cessation rates between the NRT and placebo groups.[14] Unexpectedly, infants born to NRT group women were 40% more likely to have unimpaired development at two years of age than infants born to women in the placebo groups[15]

For peer review only - http://bmjopen.bmj.com/site/about/guidelines.xhtml

(OR 1.40, 95% CI: 1.05-1.86, p=0.023) and there was a dose-response relationship between adherence to NRT and impairment-free infant development.[15] It seems implausible that this effect would be due to nicotine having a direct beneficial effect on the fetus, and there is a possibility that the observed effect might be due to differences in unmeasured characteristics between the participants in the NRT group and those in the placebo group. However, as NRT had a substantial impact on cessation in early pregnancy, we hypothesise that the better developmental outcomes in NRT group infants and the dose-response relationship between increasing NRT use and these might be explained by smoking cessation during pregnancy caused by NRT use. Consequently, we present secondary analyses of data from the same trial to investigate whether the absence of infants' developmental impairments at two years was associated with maternal smoking status measured at different points in the trial.

METHODS

Study participants

We used data from the Smoking, Nicotine and Pregnancy (SNAP) trial, a placebo-randomised -controlled trial of NRT for smoking cessation in pregnant women[14]. The trial recruited 1,050 participants aged 16-45 years between 12 and 24 weeks gestation who smoked at least ten cigarettes a day before pregnancy and at least five cigarettes a day during pregnancy, with exhaled carbon monoxide (CO) readings of ≥8ppm. Participants were randomised to receive up to an 8-week course of either 15mg per 16 hours of NRT patches or visually-identical placebo. For safety reasons, women were instructed that if they smoked at all whilst using patches, the NRT should be stopped. Participants set a target quit date and were followed up one month after this, during hospital admission for delivery or as soon as possible afterwards, and at 24 months after delivery.

For peer review only - http://bmjopen.bmj.com/site/about/guidelines.xhtml

Maternal baseline data and smoking behaviour measures

Baseline data included maternal date of birth, age on leaving full time education, number of cigarettes smoked per day prior to and during pregnancy, body mass index (BMI), the heaviness of smoking index (HSI) - a measure of nicotine dependence which is a six-point scale derived from the time to first cigarette after waking and the number of cigarettes smoked daily[16]. Other data included were previous use of NRT in the current pregnancy and partners' smoking status.

Self-reported smoking data were obtained one month after the target quit date and at delivery. Self-reported smoking abstinence at one-month after the quit date was validated by exhaled carbon monoxide (CO) measurements below 8ppm while abstinence at delivery was validated by CO measurements and saliva cotinine (COT) concentrations below 10ng/ml. Carbon monoxide is exhaled from the breath after smoking cigarettes and was used to confirm abstinence within the preceding 24 hours. Salivary cotinine on the other hand was used to determine smoking exposure within the last 7 days[17]. No data on tobacco smoke exposure were collected between the one-month follow-up date and delivery. Self-reported data on the use of nicotine patches were collected one month after the target quit date and at delivery; adherence to NRT have been reported elsewhere[14].

Infant outcome measures

Birth status (live or still birth), gestational age at birth, birth weight, singleton or multiple birth, sex and gestational age at birth were recorded at delivery and have been reported elsewhere[14]. The presence of infant developmental impairment at 24 months was determined using questionnaire responses from participants or from healthcare professionals when there was no participant response; full details have been described previously[15]. Participants reported on their infants' development as assessed within the 5 domains of the Ages and Stages Questionnaire edition 3 (ASQ-3): communication, gross motor, fine motor, problem solving and personal-social skills.[15] Participant questionnaires (PQ2) were posted out at 24 months and if no response was received, health

For peer review only - http://bmjopen.bmj.com/site/about/guidelines.xhtml

professional ones were dispatched to non-respondents' family physicians. Health professional questionnaire (HPQ) items were consistent with domains in participant questionnaires and were designed to be completed with reference to medical or health visitor records. Health professionals completing these questionnaires required relatively little knowledge of the patient. However if they were unable to complete the questionnaire, they were asked to forward these to children's' health visitors (HV)[18]. Scores from the five ASQ-3 domains and responses to 'non-domain' ASQ-3 items in the participant questionnaires were used with established norms to categorise infants as having 'developmental impairment' or 'no developmental impairment'. Infants with only HPQ responses were considered to have no developmental impairment when the responses from all questions indicated no potential developmental issues.[15] As shown in the main study, 88% of study participants returned a PQ2 (participant questionnaire) or HPQ (health professional questionnaire). The proportion of participants assessed using the PQ2 or HPQ were similar in the treatment arm and placebo arm. Approximately two-thirds of participants completed and returned the PQ2, while the HPQ was the source of data for children of the remaining participants.

Measures of tobacco smoke exposure

As outlined above, smoking behaviour measures were collected at randomisation, one month after a target quit date and at the end of pregnancy. From these data, we derived the following categories to represent different levels of tobacco smoke exposure during pregnancy:

i) Number of cigarettes smoked daily during pregnancy, at randomisation

ii) Heaviness of smoking index (HSI) at randomisation, categorised as high (4-6) or low (0-3)

iii) Women who reported not smoking between their target quit date and one month, validated using exhaled CO.

iv) Women who reported not smoking between their target quit date and the end of pregnancy or shortly afterwards, validated by either or both of exhaled CO and saliva

For peer review only

For peer review only - http://bmjopen.bmj.com/site/about/guidelines.xhtml

cotinine at the end of pregnancy and also by exhaled CO at one month after their quit date.

As all trial participants smoked at time of randomisation, exposure category (ii) was intended to dichotomise those who smoked more heavily and less heavily in the first weeks of pregnancy prior to study randomisation. For categories (iii) and (iv), we hypothesised that those demonstrating validated abstinence at both one month and the end of pregnancy (category iv) would have a lower overall tobacco smoke exposure than those for whom abstinence was only validated at one month after their quit date. However, it should be noted that no biochemical test can validate smoking cessation for a prolonged period and some women in either group may have smoked for a time between baseline / study enrolment and one month or the end of pregnancy.

Analyses

Maternal and infant characteristics were analysed by study arm, for participants who had singleton live births and 2-year data on infant developmental impairment. Summary statistics were represented as number (%), mean (SD) and median (IQR) for categorical, normal continuous and non-normal continuous variables respectively. Univariable logistic regression investigated the association between 2-year developmental outcomes and different measures of maternal tobacco smoke exposure such as baseline number of cigarettes smoked daily in pregnancy, the heaviness of smoking index during pregnancy, maternal smoking abstinence at some point during pregnancy (measured one month after the participants' set quit date) and maternal smoking abstinence during the latter stages of pregnancy (measured at delivery). In multivariable analyses, we adjusted the univariate models for baseline characteristic variables and also controlled for variables that could confound the association between maternal smoking and infant developmental impairment. Potential confounders included in the multivariable analyses were: maternal age, age at which full time education was completed, maternal BMI, partner's smoking status, infant birth weight, gestational age at birth and study arm (NRT or placebo). Confounder selection was done using the

For peer review only - http://bmjopen.bmj.com/site/about/guidelines.xhtml

change-in-estimate criteria [19], and any covariate which changed the effect size of the univariate exposure-outcome model by 10% was considered an important confounder and included in the fully-adjusted model. All analyses were performed using Stata 14. Significant associations were defined at the p≤0.05 significance level.

Patient and Public Involvement

The original SNAP study had a PPI representative who provided PPI insights for the Trial Steering Committee.

RESULTS

From 1,050 pregnant smokers enrolled in the SNAP trial, there were 12 women with twin pregnancies, 14 participants who had fetal deaths, 4 lost to follow-up and 10 who withdrew consent. Of 1,010 women with singleton live births, data on developmental impairment was available for 884 infants. Analyses therefore included maternal and 2-year records of these 884 infants (Figure 1). The self-reported median (IQR) number of daily cigarettes smoked by women prior to pregnancy was 19 (10-60). At the time of recruitment into the study, the reported median (IQR) number of cigarettes smoked daily in pregnancy was 15 (10-20). Of the infants born to participants in the study, 52% were males and less than 10% had low birth weight. Developmental impairment was present in 12.7% of these children. (n=112).

Study participants and singleton infants with 2-year outcome data had similar baseline characteristics to participants reported in the full trial cohort[15]. Aside from the fact that participants who were lost to follow-up had higher mean IMD (Index of multiple deprivation) scores implying that they lived in more deprived areas than participants who had data up to 2 years, there were only minor differences in the baseline characteristics between the participants with 2-year data and those who were lost to follow-up[15].

For peer review only - http://bmjopen.bmj.com/site/about/guidelines.xhtml

Figure 1.

Maternal and infant characteristics

The baseline characteristics of the women and children included in the analyses were found to be similar in participants randomised to the NRT or placebo study arm (presented in table 1). The majority of participants included in the study (72.4%) were less than 30 years old; the median BMI (IQR) at randomisation into the study was 25.9 (22.2-30.7). The average age of leaving full time education was 16.3 years. We used the 'heaviness of smoking index' as a measure of the level of maternal nicotine dependence and 65% of women in the study had low levels of nicotine dependence prior to being recruited in the study. More than two-thirds of the study participants had partners who smoked (67.9%). Overall, 18% of women were validated as abstinent from smoking at some point during pregnancy (measured one month after their target quit date) and 8.9% remained abstinent at the latter stages of pregnancy until delivery. There was a statistically significant difference in smoking cessation outcomes between participants in the NRT and placebo arms at the one-month follow-up but not at delivery.

For peer review only - http://bmjopen.bmj.com/site/about/guidelines.xhtml

Table 1. Maternal and infant characteristics, by study arm (n=884)

	Unit	NRT arm (n=443)	Placebo-arm (n=441)	p-value
		**Maternal characteristics**		
**Maternal age (years) at pregnancy**				
<20		72 (16.25)	64 (14.51)	
20-24		142 (32.05)	150 (34.01)	
25-29	n (%)	110 (24.83)	102 (23.13)	0.275
30-34		64 (14.45)	84 (19.05)	
35-39		44 (9.93)	30 (6.80)	
>40		11 (2.48)	11 (2.49)	
**Maternal BMI [kg/m^2]** (n=847)	median(IQR)	25.71 (22.2-30.7)	26.32 (22.5-30.8)	0.042
**Index of multiple deprivation score (IMD)**	Mean (SD)	32.11 (16.83)	32.29 (16.84)	0.754
**Maternal age (years) of leaving full time education (n=872)**	mean (SD)	16.17 (1.36)	16.32 (1.70)	0.950
**Partner's smoking status**				
Non smoker		108 (24.38)	106 (24.04)	0.962
Smoker	n (%)	301 (67.95)	299 (67.80)	
No data on partner's smoking status		34 (7.67)	36 (8.16)	
**Heaviness of smoking index (HSI) at study randomisation**				
Low index (0-3)	n (%)	284 (64.11)	292 (66.21)	0.511
High index (4-6)		159 (35.89)	149 (33.79)	
**Daily number of cigarettes smoked at study randomisation**	median (IQR)	13 (10-20)	15 (10-20)	0.799
**Smoking cessation measured during pregnancy (at 1 month follow-up)**				
Did not cease smoking	n (%)	341 (76.98)	384 (87.07)	<0.0001
Ceased smoking		102 (23.02)	57 (12.93)	
**Maternal smoking cessation measured at time of delivery**				
Did not cease smoking	n (%)	397 (89.62)	408 (92.52)	0.131
Ceased smoking		46 (10.38)	33 (7.48)	
		**Infant characteristics**		
**Infant sex**				
Female	n (%)	217 (48.98)	205 (46.49)	0.457
male		226 (51.02)	236 (53.51)	
**Birth weight**				
<2.5kg		48 (10.84)	37 (8.39)	
2.5-3.0kg	n (%)	111 (25.06)	104 (23.58)	0.534
3.0-3.5kg		161 (36.34)	174 (39.46)	
>3.5kg		123 (27.77)	126 (28.57)	
**Gestational age at birth (weeks)**	median (IQR)	39.5 (2.1)	39.5 (2.2)	

For peer review only

For peer review only - http://bmjopen.bmj.com/site/about/guidelines.xhtml

Analyses of the associations between different measures of maternal tobacco smoke exposure and infant developmental impairment found no statistically significant relationship between maternal smoking exposure and infant developmental impairment at age 2 (Table 2). The odds ratio for developmental impairment in infants of mothers with validated smoking cessation at some point during pregnancy (measured at the one-month follow-up) and in infants of mothers with validation cessation at delivery, were 0.99 (95% CI 0.59-1.66) and 1.55 (95% CI 0.84-2.87) respectively. Adjustment for maternal and infant baseline characteristics and covariates did not significantly alter the association between maternal smoking and infant development. Table 2 shows the unadjusted odds ratios with the different measures of cigarette smoke exposure, as well as the odds-ratios adjusted by maternal and infant baseline characteristics.

Table 2. Multivariate models of the association between maternal tobacco smoke exposure in pregnancy and child developmental impairment at 2 years (analyses restricted to 884 mothers with measures of infant 2-year developmental outcome)

	**Odds ratio (95% CI) for developmental impairment at age 2**	
	Unadjusted	Adjusted₸
**Daily number of cigarettes smoked at study randomisation**	1.01 (0.98-1.04)	1.00 (0.97-1.03)
**Maternal heaviness of smoking index (HSI) at study randomisation (high vs low)**	1.30 (0.87-1.95)	1.26 (0.84-1.90)
**Maternal smoking cessation in pregnancy (measured 1 month after randomisation)**	0.99 (0.59-1.66)	1.03 (0.61-1.75)
**Maternal abstinence from smoking during latter stages of pregnancy (y/n)**	1.55 (0.84-2.87)	1.53 (0.82-2.87)

₸ Adjusted for maternal age, gestational age at birth and infant birthweight

For peer review only

For peer review only - http://bmjopen.bmj.com/site/about/guidelines.xhtml

DISCUSSION

Summary of principal findings

We found no associations between maternal smoking status at different points during pregnancy and infants' developmental impairment at two years. Consequently, there is no evidence to support the hypothesis that the better infant development observed within infants born to women who were randomised to NRT in the Smoking, Nicotine and Pregnancy (SNAP) trial resulted from smoking cessation induced by nicotine patch use.

Strengths and limitations

Data for this study are from the largest randomised controlled trial of NRT in pregnancy in which participants, health care and research staff were masked to treatment allocation[14]. Follow-up and outcome ascertainment rates were high and there were only minor differences in characteristics of participants lost to follow up and those remaining in the study at 2 years[15]. It remains the only trial of a smoking cessation intervention in pregnancy to monitor infant outcomes; as such it is the only source of data which could be used to investigate the study hypothesis.

The ASQ-3 is a well-validated screening tool which has a sensitivity of 100% and specificity of 93% for detecting severe developmental delay at 24 months[20]. While some study infants may have been falsely identified as developmentally impaired, there is no reason to believe that such ASQ-3 false positives would have occurred more frequently in infants of mothers who smoked in pregnancy. Whist developmental impairment was assessed in some infants using the participant questionnaire, others had the health professional questionnaire as the main source of data on impairment. Although this consequently implies a non-standardised assessment of the outcome in all infants, findings from the main study had shown that the proportion of participants assessed with either the PQ2 or the HPQ were similar in the treatment and placebo arms. Also, there were similarities in the

For peer review only - http://bmjopen.bmj.com/site/about/guidelines.xhtml

baseline characteristics and birth outcomes of participants who returned the PQ2 and those for whom the HPQs were completed.

Although we adjusted for some potentially confounding factors which are known to be associated with infant development such as maternal socioeconomic status[21], maternal education[22], low birth weight[23], maternal exposure to passive smoking[24] and maternal obesity[25] ,we were unable to adjust for those on which we had no data, such as maternal nutrition[26], depressive illness[27], stress and anxiety[28] and alcohol consumption[29] which could also potentially influence infant development but which were not measured in the SNAP trial. There remains therefore, the potential for residual confounding.

We undertook secondary analyses of randomised controlled trial data and the original sample size was intended to have sufficient power only for detecting differences in validated smoking cessation rates at the end of pregnancy. Analyses presented here may not have sufficient power to demonstrate associations between smoking and infant development; however, one would expect to find one or more such associations if, the relationship between NRT use and infant development which was demonstrated within the same data, were principally explained by smoking cessation caused by NRT.

There may have been some misattribution of smoking status. Measures of smoking status used were biochemically-validated at two different time points in pregnancy. Validation with carbon monoxide at the earlier time point could only eliminate smoking within the previous 24 hours[30]; and at delivery, with saliva could only do so for the previous seven days[17]. Consequently, some participants may have relapsed or smoked occasionally between these times[31]; however we believe it is reasonable to assume that women who were validated as not smoking both at the end of pregnancy and at one month after randomisation would have lower overall tobacco smoke exposure than those who were not. As recruitment into the study occurred between 12 and 24 weeks gestation, the timing of smoking status measurements in relation to gestation will have differed

For peer review only - http://bmjopen.bmj.com/site/about/guidelines.xhtml

For peer review only

between participants but there is no evidence that smoking is more or less harmful at any point in pregnancy. Although these were the best available data for analyses, the assumptions outlined above need to be considered when interpreting study findings.

As most trial participants continued to smoke, analyses might have been more illuminating if data on the intensity of women's smoking during pregnancy had been available. For example, if exhaled CO measurements or numbers of cigarettes smoked daily in pregnancy had been available for non-abstinent trial participants, these data could have been used to investigate whether smoking intensity in pregnancy was associated infant development. Increasing smoking intensity, reflected by exhaled CO levels, is associated with reduced infant birth weights[32], so an association with development seems possible.

Our analyses did not incorporate postnatal environmental cigarette smoke exposure which may have an independent influence on infant development[33]. We know that there is continued maturation of the connections between brain regions after birth, with intense brain white matter myelination in the first post-natal months which becomes progressively less rapid through toddlerhood until young adulthood[34]. Additionally, postnatal exposure to environmental tobacco is associated with emotional and conduct behavioural problems in school aged children[35] and so may have an aetiological role in infant developmental impairment. As maternal prenatal and postnatal smoking are strongly correlated, this would increase the likelihood of finding associations between smoking in pregnancy and infant development; as none were found there is no suggestion of a large impact on findings. Nonetheless, there is a need to account for environmental cigarette smoke exposure in future studies.

Comparison with literature

As this is an original analysis in a unique trial database there is very little literature with which study findings can be compared. Previous observational studies have found prenatal tobacco exposure to

For peer review only - http://bmjopen.bmj.com/site/about/guidelines.xhtml

be associated with offspring adverse consequences including behavioural[36] and hyperactivity[37 38] problems, language and reading deficits[37], conduct disorders[39], newborn basic perceptual skills[40] and intellectual impairment in childhood[41] and later life[42]. However, other studies which adjusted for potentially confounding factors such as maternal education [43 44] and parental socioeconomic status[45]have not. Consistent with these latter studies, our findings found no evidence that the improved developmental outcomes in infants of pregnant smokers who used NRT in the SNAP trial[15] were explained by smoking cessation. As pre-clinical studies show that nicotine is neurotoxic and can adversely affect the developing central nervous system[11 12], it seems unlikely that these better outcomes are due to a direct protective effect of NRT on the developing fetus. Also, compliance rates in the NRT patch and placebo study arms were low in the SNAP trial (7.2% and 2.8% compliance respectively, at one month). Perhaps, it is possible that nicotine used in pregnancy has no impact on infants' developmental outcomes and the apparently protective effect of NRT on developmental outcomes observed in the SNAP trial occurred by chance.

CONCLUSION

We found no evidence that the better 2- year developmental outcomes in offspring of pregnant women randomised to NRT patch use in the Smoking, Nicotine and Pregnancy (SNAP) trial, was due to the smoking cessation caused by NRT. Future research which takes account of women's intensity of smoking in pregnancy as well as important maternal confounders and postnatal exposures, is needed to investigate how NRT use in pregnancy may exert this effect on infant development.

For peer review only - http://bmjopen.bmj.com/site/about/guidelines.xhtml

ACKNOWLEDGEMENTS

We gratefully acknowledge the SNAP trial team members. In addition to listed authors, the complete trial team includes: Investigators : Kim Watts, Jim Thornton, Jo Leonardi-Bee, John Britton, Paul Aveyard, Michael Coughtrie, Christine Godfrey, Clare Mannion and Neil Marlow. Research staff: Janet Brown, Yvette Davis, Anne Dickinson, Caroline Dixon, Fiona Holloway, Joanne Lakin, Jayne Platts, Farzana Rashid, Amanda Redford, Cara Taylor. Principal investigators (in recruiting centres): Jonathan Allsop, Simon Cunningham, Karen Glass, Vince Hall, Khaled Ismail, Margaret Ramsay. Midwife leads (in recruiting centres): Sheena Appleby. Denise Bailey, Linda Gustard, Emma Haworth, Grace Hopps, Amanda Lindley, Chris Kettle, Colleen Pearce, Dymphna Sexton-Bradshaw, Julia Savage, Sandra Smith, Sheila Taylor, Alison Witham. Primary Care Trust & NHS Stop Smoking Services' Staff : Barbara Brady, Michelle Battlemuch, Wendy Dudley, Rochelle Edwards, Lorraine Frith, Indu Hari, Catriona Holden, Linda Hoskyns, Paul Jackson, Giri Rajaratnam, Deborah Richardson, Lucy Wade, Maureen Whittaker. QMC Pharmacy: Bernie Cook, Sheila Hodgson (Lead pharmacist), Lisa Humphries, Bernie Sanders (Qualified Person). University of Nottingham Clinical Trials Unit: Dan Simpkins. University of Dundee: Sheila Sharp.

COMPETING INTERESTS

The authors have no conflicts of interest relevant to this study.

FUNDING

Views and opinions expressed are those of the authors and do not necessarily reflect those of the National Institute for Health Research (NIHR) Health Technology Assessment (HTA) Programme, the NIHR, the National Health Service (NHS) or the English Department of Health. This trial was supported by a grant from the NIHR HTA Programme (project number 06/07/01). BI's clinical academic lectureship is fully funded by the NIHR. TC, LV, SC and, SL, and TC are members of the UK Centre for Tobacco and Alcohol Studies (UKCTAS). TC, LV, SC and JT are members of the NIHR School for Primary care Research. Professor Coleman is a National Institute for Health Research Senior Investigator.

For peer review only

DETAILS OF ETHICS APPROVAL

National research ethical approval for the SNAP trial was granted by Oxfordshire Research Ethics Committee A, and additional local approvals for each recruitment centre and Clinical Trial Authorisation (CTA) approval was from the MHRA (CTA number: 03057/0002/001-0001). All participants gave written informed consent, including access to medical records for maintaining contact and for the follow-up of their child's health status.

AUTHOR STATEMENT

BI had full access to all of the data in the study and takes responsibility for the integrity of the data and the accuracy of the analyses. BI also wrote the first draft of the manuscript. LV contributed to the initial stages of data organisation, study concept and design, and proofread and approved the full manuscript prior to submission. TC contributed to the study hypothesis and design, data interpretation and analyses, assisted with writing up the "introduction" section and proofread and approved the final manuscript prior to submission. JT, SL and SC contributed to the data interpretation and analyses of this study as well as revision of the manuscript prior to submission.

DATA SHARING STATEMENT

The study protocol for the SNAP trial is publicly available online. Requests for access to de-identified individual participant data that underlie the results reported in this article should be made to the authors of the original study.

For peer review only

For peer review only - http://bmjopen.bmj.com/site/about/guidelines.xhtml

Figure legends

Figure 1. Flow diagram of study participants

Participants with twin pregnancies (n=12), fetal deaths (n=14), loss to follow-up (n=4) and those who withdrew consent (n=10) were excluded from the study analyses. Among participants with singleton live births, there were no data on 2-year infant outcomes for 126 participants, so these participants were also excluded from the analyses.

For peer review only

For peer review only - http://bmjopen.bmj.com/site/about/guidelines.xhtml

REFERENCES

1. Smoking and the young. A report of a working party of the Royal College of Physicians. London: RCP, 1992.

2. Mohsin M, Bauman AE. Socio-demographic factors associated with smoking and smoking cessation among 426,344 pregnant women in New South Wales, Australia. *Bmc Public Health* 2005;5 doi: Artn 138 Doi 10.1186/1471-2458-5-138

3. Jensen KE, Jensen A, Nohr B, et al. Do pregnant women still smoke? A study of smoking patterns among 261,029 primiparous women in Denmark 1997-2005. *Acta Obstetricia Et Gynecologica Scandinavica* 2008;87(7):760-67. doi: Doi 10.1080/00016340802179814

4. Centers for Disease Control and Prevention. Smoking during pregnancy--United States, 1990-2002. *MMWR Morb Mortal Wkly Rep* 2004;39(53):4.

5. McAndrew F, Thompson J, Fellows L, et al. Infant Feeding Survey 2010: Health and Social Care Information Centre, 2012.

6. Oncken CA, Dietz PM, Tong VT, et al. Prenatal tobacco prevention and cessation interventions for women in low- and middle-income countries. *Acta Obstet Gynecol Scand* 2010;89(4):442-53. doi: 10.3109/00016341003678450 [published Online First: 2010/03/20]

7. World Health Organization. Report on the Global Tobacco Epidemic 2008—the mpower package. Geneva: World Health Organization, 2008.

8. Chamberlain C, O'Mara-Eves A, Oliver S, et al. Psychosocial interventions for supporting women to stop smoking in pregnancy. *Cochrane Database Syst Rev* 2013;10:CD001055. doi: 10.1002/14651858.CD001055.pub4

9. Stead LF, Perera R, Bullen C, et al. Nicotine replacement therapy for smoking cessation. *Cochrane Database Syst Rev* 2012;11:CD000146. doi: 10.1002/14651858.CD000146.pub4

10. Dhalwani NN, Szatkowski L, Coleman T, et al. Prescribing of nicotine replacement therapy in and around pregnancy: a population-based study using primary care data. *Br J Gen Pract* 2014;64(626):e554-60. doi: 10.3399/bjgp14X681361

11. Dempsey DA, Benowitz NL. Risks and benefits of nicotine to aid smoking cessation in pregnancy. [Review] [340 refs]. *Drug Safety* 2001;24(4):277-322.

12. Benowitz N, Dempsey D. Pharmacotherapy for smoking cessation during pregnancy. *Nicotine & Tobacco Research* 2004;6 Suppl 2:S189-202. doi: 10.1080/14622200410001669169 [published Online First: 2004/06/19]

13. Benowitz NL, Dempsey DA, Goldenberg RL, et al. The use of pharmacotherapies for smoking cessation during pregnancy. *Tobacco Control* 2000;9 Suppl 3:III91-III94.

14. Coleman T, Cooper S, Thornton JG, et al. A randomized trial of nicotine-replacement therapy patches in pregnancy. *New England Journal of Medicine* 2012;366(9):808-18. doi: http://dx.doi.org/10.1056/NEJMoa1109582

15. Cooper S, Taggar J, Lewis S, et al. Effect of nicotine patches in pregnancy on infant and maternal outcomes at 2 years: follow-up from the randomised, double-blind, placebo-controlled SNAP trial. *The Lancet Respiratory Medicine* 2014;2(9):728-37. doi: http://dx.doi.org/10.1016/S2213-2600(14)70157-2

For peer review only - http://bmjopen.bmj.com/site/about/guidelines.xhtml

16. Heatherton TF, Kozlowski LT, Frecker RC, et al. Measuring the heaviness of smoking: using self-reported time to the first cigarette of the day and number of cigarettes smoked per day. *British Journal of Addiction* 1989;84(7):791-99.

17. Montalto NJ, Wells WO. Validation of Self-Reported Smoking Status Using Saliva Cotinine: A Rapid Semiquantitative Dipstick Method. *Cancer Epidemiology Biomarkers & Prevention* 2007;16(9):1858. doi: 10.1158/1055-9965.EPI-07-0189

18. Protocol for the SNAP (Smoking Nicotine and Pregnancy) Trial Final version 70 2009.

19. Lee PH. Is a Cutoff of 10% Appropriate for the Change-in-Estimate Criterion of Confounder Identification? *Journal of Epidemiology* 2014;24(2):161-67. doi: 10.2188/jea.JE20130062

20. Gollenberg A, Lynch C, Jackson L, et al. Concurrent validity of the parent-completed Ages and Stages Questionnaires, 2nd Ed. with the Bayley Scales of Infant Development II in a low-risk sample. *Child Care Health and Development* 2010;36(4):485-90. doi: 10.1111/j.1365-2214.2009.01041.x

21. Ronfani L, Brumatti L, Mariuz M, et al. The Complex Interaction between Home Environment, Socioeconomic Status, Maternal IQ and Early Child Neurocognitive Development: A Multivariate Analysis of Data Collected in a Newborn Cohort Study. *Plos One* 2015;10(5) doi: 10.1371/journal.pone.0127052

22. Carneiro P, Meghir C, Parey M. MATERNAL EDUCATION, HOME ENVIRONMENTS, AND THE DEVELOPMENT OF CHILDREN AND ADOLESCENTS. *Journal of the European Economic Association* 2013;11:123-60. doi: 10.1111/j.1542-4774.2012.01096.x

23. Stein R, Siegel M, Bauman L. Are children of moderately low birth weight at increased risk for poor health? A new look at an old question. *Pediatrics* 2006;118(1):217-23. doi: 10.1542/peds.2005-2836

24. Jorge JG, Botelho C, Silva AM, et al. Influence of passive smoking on learning in elementary school. *J Pediatr (Rio J)* 2016;92(3):260-7. doi: 10.1016/j.jped.2015.08.010

25. Wylie A, Sundaram R, Kus C, et al. Maternal Prepregnancy Obesity and Achievement of Infant Motor Developmental Milestones in the Upstate KIDS Study. *Obesity* 2015;23(4):907-13. doi: 10.1002/oby.21040

26. Marques A, O'Connor T, Roth C, et al. The influence of maternal prenatal and early childhood nutrition and maternal prenatal stress on offspring immune system development and neurodevelopmental disorders. *Frontiers in Neuroscience* 2013;7 doi: 10.3389/fnins.2013.00120

27. Ibanez G, Bernard J, Rondet C, et al. Effects of Antenatal Maternal Depression and Anxiety on Children's Early Cognitive Development: A Prospective Cohort Study. *Plos One* 2015;10(8) doi: 10.1371/journal.pone.0135849

28. Tarabulsy G, Pearson J, Vaillancourt-Morel M, et al. Meta-Analytic Findings of the Relation Between Maternal Prenatal Stress and Anxiety and Child Cognitive Outcome. *Journal of Developmental and Behavioral Pediatrics* 2014;35(1):38-43.

29. Knudsen A, Skogen J, Ystrom E, et al. Maternal pre-pregnancy risk drinking and toddler behavior problems: the Norwegian Mother and Child Cohort Study. *European Child & Adolescent Psychiatry* 2014;23(10):901-11. doi: 10.1007/s00787-014-0588-x

For peer review only - http://bmjopen.bmj.com/site/about/guidelines.xhtml

30. Secker-Walker RH, Vacek PM, Flynn BS, et al. Exhaled carbon monoxide and urinary cotinine as measures of smoking in pregnancy. *Addict Behav* 1997;22(5):671-84.

31. Parazzini F, Davoli E, Rabaiotti M, et al. Validity of self-reported smoking habits in pregnancy: a saliva cotinine analysis. *Acta Obstet Gynecol Scand* 1996;75(4):352-4.

32. Reynolds CME, Egan B, Kennedy RA, et al. The implications of high carbon monoxide levels in early pregnancy for neonatal outcomes. *European Journal of Obstetrics and Gynecology and Reproductive Biology* 2019;233:6-11. doi: 10.1016/j.ejogrb.2018.11.020

33. Yolton K, Dietrich K, Auinger P, et al. Exposure to environmental tobacco smoke and cognitive abilities among U.S. children and adolescents. *Environmental health perspectives* 2005;113(1):98-103. doi: 10.1289/ehp.7210 [published Online First: 10/07]

34. Dubois J, Dehaene-Lambertz G, Kulikova S, et al. THE EARLY DEVELOPMENT OF BRAIN WHITE MATTER: A REVIEW OF IMAGING STUDIES IN FETUSES, NEWBORNS AND INFANTS. *Neuroscience* 2014;276:48-71. doi: 10.1016/j.neuroscience.2013.12.044

35. Chastang J, Baiz N, Cadwalladder J, et al. Postnatal Environmental Tobacco Smoke Exposure Related to Behavioral Problems in Children. *Plos One* 2015;10(8) doi: 10.1371/journal.pone.0133604

36. Carter S, Paterson J, Gao W, et al. Maternal smoking during pregnancy and behaviour problems in a birth cohort of 2-year-old Pacific children in New Zealand. *Early Hum Dev* 2008;84(1):59-66. doi: 10.1016/j.earlhumdev.2007.03.009 [published Online First: 2007/05/17]

37. Naeye RL, Peters EC. Mental development of children whose mothers smoked during pregnancy. *Obstet Gynecol* 1984;64(5):601-7.

38. Langley K, Holmans PA, van den Bree MB, et al. Effects of low birth weight, maternal smoking in pregnancy and social class on the phenotypic manifestation of Attention Deficit Hyperactivity Disorder and associated antisocial behaviour: investigation in a clinical sample. *BMC Psychiatry* 2007;7:26. doi: 10.1186/1471-244X-7-26 [published Online First: 2007/06/20]

39. Wakschlag LS, Lahey BB, Loeber R, et al. Maternal smoking during pregnancy and the risk of conduct disorder in boys. *Arch Gen Psychiatry* 1997;54(7):670-6.

40. Key AP, Ferguson M, Molfese DL, et al. Smoking during pregnancy affects speech-processing ability in newborn infants. *Environ Health Perspect* 2007;115(4):623-9. doi: 10.1289/ehp.9521 [published Online First: 2006/11/28]

41. Olds DL, Henderson CR, Tatelbaum R. Intellectual impairment in children of women who smoke cigarettes during pregnancy. *Pediatrics* 1994;93(2):221-7.

42. Mortensen EL, Michaelsen KF, Sanders SA, et al. A dose-response relationship between maternal smoking during late pregnancy and adult intelligence in male offspring. *Paediatr Perinat Epidemiol* 2005;19(1):4-11. doi: 10.1111/j.1365-3016.2004.00622.x

43. Batty GD, Der G, Deary IJ. Effect of maternal smoking during pregnancy on offspring's cognitive ability: empirical evidence for complete confounding in the US national longitudinal survey of youth. *Pediatrics* 2006;118(3):943-50. doi: 10.1542/peds.2006-0168

For peer review only

For peer review only - http://bmjopen.bmj.com/site/about/guidelines.xhtml

44. Breslau N, Paneth N, Lucia VC, et al. Maternal smoking during pregnancy and offspring IQ. *Int J Epidemiol* 2005;34(5):1047-53. doi: 10.1093/ije/dyi163 [published Online First: 2005/08/05]

45. Roza SJ, Verhulst FC, Jaddoe VW, et al. Maternal smoking during pregnancy and child behaviour problems: the Generation R Study. *Int J Epidemiol* 2009;38(3):680-9. doi: 10.1093/ije/dyn163 [published Online First: 2008/09/04]

For peer review only

For peer review only - http://bmjopen.bmj.com/site/about/guidelines.xhtml

Figure 1. Flow diagram of study participants

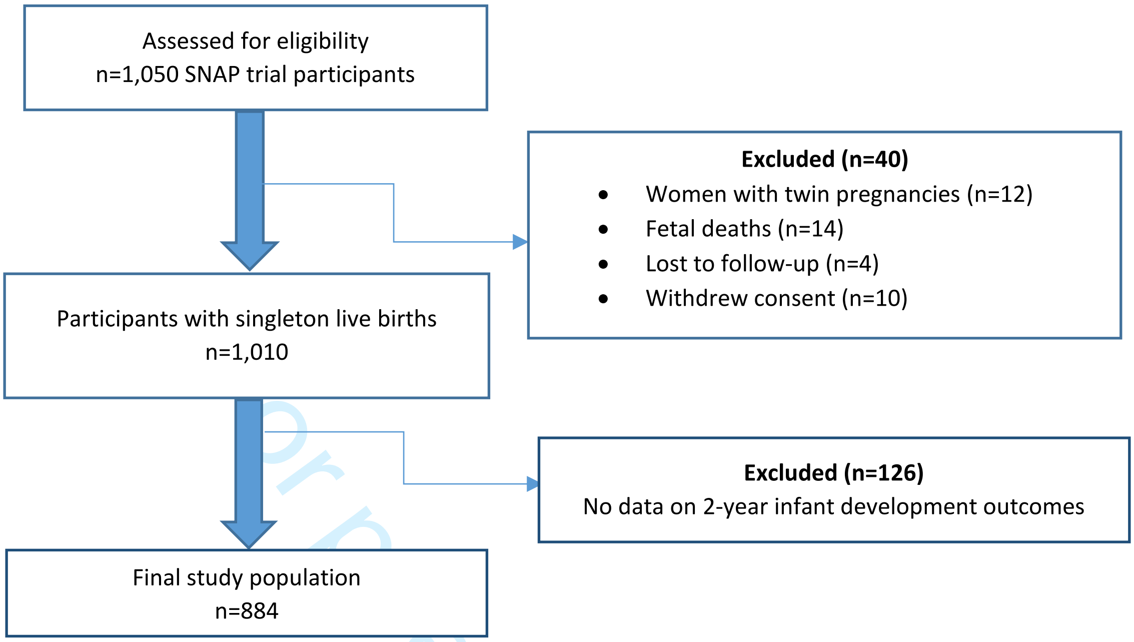

For peer review only

STROBE 2007 (v4) checklist of items to be included in reports of observational studies in epidemiology*
Checklist for cohort, case-control, and cross-sectional studies (combined)

Section/Topic	Item #	Recommendation	Reported on page #
**Title and abstract**	1	(*a*) Indicate the study's design with a commonly used term in the title or the abstract	1
		(*b*) Provide in the abstract an informative and balanced summary of what was done and what was found	2
**Introduction**			
Background/rationale	2	Explain the scientific background and rationale for the investigation being reported	4
Objectives	3	State specific objectives, including any pre-specified hypotheses	5
**Methods**			
Study design	4	Present key elements of study design early in the paper	5
Setting	5	Describe the setting, locations, and relevant dates, including periods of recruitment, exposure, follow-up, and data collection	5
Participants	6	(*a*) *Cohort study*—Give the eligibility criteria, and the sources and methods of selection of participants. Describe methods of follow-up<br>*Case-control study*—Give the eligibility criteria, and the sources and methods of case ascertainment and control selection. Give the rationale for the choice of cases and controls<br>*Cross-sectional study*—Give the eligibility criteria, and the sources and methods of selection of participants	5
		(*b*) *Cohort study*—For matched studies, give matching criteria and number of exposed and unexposed<br>*Case-control study*—For matched studies, give matching criteria and the number of controls per case	
Variables	7	Clearly define all outcomes, exposures, predictors, potential confounders, and effect modifiers. Give diagnostic criteria, if applicable	5,6,7
Data sources/ measurement	8*	For each variable of interest, give sources of data and details of methods of assessment (measurement). Describe comparability of assessment methods if there is more than one group	*5,6,7*
Bias	9	Describe any efforts to address potential sources of bias	5,6
Study size	10	Explain how the study size was arrived at	
Quantitative variables	11	Explain how quantitative variables were handled in the analyses. If applicable, describe which groupings were chosen and why	5,6,7
Statistical methods	12	(*a*) Describe all statistical methods, including those used to control for confounding	8
		(*b*) Describe any methods used to examine subgroups and interactions	n/a
		(*c*) Explain how missing data were addressed	9
		(*d*) *Cohort study*—If applicable, explain how loss to follow-up was addressed<br>*Case-control study*—If applicable, explain how matching of cases and controls was addressed	

For peer review only

		Cross-sectional study—If applicable, describe analytical methods taking account of sampling strategy	
		(*e*) Describe any sensitivity analyses	
**Results**			
Participants	13*	(a) Report numbers of individuals at each stage of study—eg numbers potentially eligible, examined for eligibility, confirmed eligible, included in the study, completing follow-up, and analysed	9
		(b) Give reasons for non-participation at each stage	9
		(c) Consider use of a flow diagram	9
Descriptive data	14*	(a) Give characteristics of study participants (eg demographic, clinical, social) and information on exposures and potential confounders	9,10,11
		(b) Indicate number of participants with missing data for each variable of interest	11
		(c) *Cohort study*—Summarise follow-up time (eg, average and total amount)	
Outcome data	15*	*Cohort study*—Report numbers of outcome events or summary measures over time	9,10,11,12
		Case-control study—Report numbers in each exposure category, or summary measures of exposure	
		Cross-sectional study—Report numbers of outcome events or summary measures	
Main results	16	(*a*) Give unadjusted estimates and, if applicable, confounder-adjusted estimates and their precision (eg, 95% confidence interval). Make clear which confounders were adjusted for and why they were included	10,11
		(*b*) Report category boundaries when continuous variables were categorized	
		(*c*) If relevant, consider translating estimates of relative risk into absolute risk for a meaningful time period	
Other analyses	17	Report other analyses done—eg analyses of subgroups and interactions, and sensitivity analyses	
**Discussion**			
Key results	18	Summarise key results with reference to study objectives	13
Limitations	19	Discuss limitations of the study, taking into account sources of potential bias or imprecision. Discuss both direction and magnitude of any potential bias	13,14
Interpretation	20	Give a cautious overall interpretation of results considering objectives, limitations, multiplicity of analyses, results from similar studies, and other relevant evidence	13,14,15
Generalisability	21	Discuss the generalisability (external validity) of the study results	15,16
**Other information**			
Funding	22	Give the source of funding and the role of the funders for the present study and, if applicable, for the original study on which the present article is based	17

*Give information separately for cases and controls in case-control studies and, if applicable, for exposed and unexposed groups in cohort and cross-sectional studies.
Note: An Explanation and Elaboration article discusses each checklist item and gives methodological background and published examples of transparent reporting. The STROBE checklist is best used in conjunction with this article (freely available on the Web sites of PLoS Medicine at http://www.plosmedicine.org/, Annals of Internal Medicine at http://www.annals.org/, and Epidemiology at http://www.epidem.com/). Information on the STROBE Initiative is available at www.strobe-statement.org.

For peer review only
